# Cellular localization of relaxin‐like gonad‐stimulating peptide expression in *Asterias rubens*: New insights into neurohormonal control of spawning in starfish

**DOI:** 10.1002/cne.24141

**Published:** 2016-11-21

**Authors:** Ming Lin, Masatoshi Mita, Michaela Egertová, Cleidiane G. Zampronio, Alexandra M. Jones, Maurice R. Elphick

**Affiliations:** ^1^ Queen Mary University of London, School of Biological & Chemical SciencesLondonUK; ^2^ Department of BiologyFaculty of Education, Tokyo Gakugei UniversityTokyoJapan; ^3^ School of Life Sciences and Proteomics Research Technology PlatformUniversity of WarwickCoventryUK

**Keywords:** echinoderm, neuropeptide, gonadotropin, gamete, spawning, ovary, mRNA in situ hybridization, RRID AB_2617214

## Abstract

Gamete maturation and spawning in starfish is triggered by a gonad‐stimulating substance (GSS), which is present in extracts of the radial nerve cords. Purification of GSS from the starfish *Patiria pectinifera* identified GSS as a relaxin‐like polypeptide, which is now known as relaxin‐like gonad‐stimulating peptide (RGP). Cells expressing RGP in the radial nerve cord of *P. pectinifera* have been visualized, but the presence of RGP‐expressing cells in other parts of the starfish body has not been investigated. Here we addressed this issue in the starfish *Asterias rubens*. An *A. rubens* RGP (AruRGP) precursor cDNA was sequenced and the A chain and B chain that form AruRGP were detected in *A. rubens* radial nerve cord extracts using mass spectrometry. Comparison of the bioactivity of AruRGP and *P. pectinifera* RGP (PpeRGP) revealed that both polypeptides induce oocyte maturation and ovulation in *A. rubens* ovarian fragments, but AruRGP is more potent than PpeRGP. Analysis of the expression of AruRGP in *A. rubens* using mRNA in situ hybridization revealed cells expressing RGP in the radial nerve cords, circumoral nerve ring, and tube feet. Furthermore, a band of RGP‐expressing cells was identified in the body wall epithelium lining the cavity that surrounds the sensory terminal tentacle and optic cushion at the tips of the arms. Discovery of these RGP‐expressing cells closely associated with sensory organs in the arm tips is an important finding because these cells are candidate physiological mediators for hormonal control of starfish spawning in response to environmental cues. J. Comp. Neurol. 525:1599–1617, 2017. © 2016 Wiley Periodicals, Inc.

**ABBREVIATIONS**
Aam RGP: *Asterias amurensis* relaxin‐like gonad‐stimulating peptide
Aja RGP: *Aphelasterias japonica* relaxin‐like gonad‐stimulating peptide
AP: Alkaline phosphatase
AruRGP: *Asterias rubens* relaxin‐like gonad‐stimulating peptide
AruRLP2: *Asterias rubens* relaxin‐type precursor
ASW: Artificial seawater
BCIP: 5‐bromo‐4‐chloro‐3′‐indolyphosphate p‐toluidine
CL: Connective tissue layer
CONR: Circumoral nerve ring
CRH: Corticotropin‐releasing hormone
DIG: Digoxygenin
Ec: Ectoneural region
EC_50_: Median effective concentration
ELH: Egg laying hormone
Ep: Epithelial layer
FSH: Follicle‐stimulating hormone
GnRH: Gonadotropin‐releasing hormone
GSS: Gonad‐stimulating substance
Hy: Hyponeural region
IGF: Insulin‐like growth factor
LH: Luteinizing hormone
ML: Muscle layer
MS: Mass spectrometry
NBT: Nitro‐blue tetrazolium
OC: Optic cushion
PBS: Phosphate‐buffered saline
PBST: PBS / 0.1% Tween‐20
PCR: Polymerase chain reaction
PFA: Paraformaldehyde
PMSF: Phenylmethylsulfonyl fluoride
Ppe: RGP *Patiria pectinifera* relaxin‐like gonad‐stimulating peptide
RG: Prelaxin‐like gonad‐stimulating peptide
Sp: Spine
SSC: Saline‐sodium citrate
Su: Sucker
TF: Tube foot
TT: Terminal tentacle

Control of reproductive maturation and function in animals is regulated by a variety of polypeptide hormones. In vertebrates, hypothalamic gonadotropin‐releasing hormone (GnRH) acts on the pituitary gland to stimulate release of follicle‐stimulating hormone (FSH) and luteinizing hormone (LH), which then act synergistically to promote gamete maturation and gonadal function (Pierce and Parsons, 1981). Gonadotropic hormones identified in invertebrates include egg‐laying hormone (ELH) in mollusks, which is related to vertebrate corticotropin‐releasing hormone (CRH) (Chiu et al., 1979; Conn and Kaczmarek, 1989), and an insulin‐like peptide that regulates egg maturation in the mosquito *Aedes aegypti* (Brown et al., 2008).

The first report of a gonadotropic substance in an invertebrate was the observation that extracts of radial nerve cords from the starfish *Asterias forbesi* induce shedding of gametes when injected in this species (Chaet and McConnaughy, 1959). The active substance was named gonad‐stimulating substance (GSS) and was characterized biochemically as a peptide hormone (Kanatani, 1979). Furthermore, antibodies to GSS were generated and used to localize its expression in the starfish *Pycnopodia helianthoides*, with immunostaining observed in the radial hemal sinus located above the radial nerve cords (Caine and Burke, 1985). However, the molecular identity of GSS was not determined until 2009, 50 years after its activity was first reported (Mita et al., 2009a). Using the Japanese starfish species *Patiria pectinifera* as an experimental system, GSS was purified and identified as a heterodimer comprising two polypeptides: A and B chains. The A and B chains are linked by two disulfide bridges, with the A chain also having a single intramolecular disulfide bridge. Furthermore, the A chain contains a cysteine motif CCxxxCxxxxxxxxC, which is a signature sequence of the insulin/insulin‐like growth factor (IGF)/relaxin superfamily (Mita et al., 2009a). More specifically, phylogenetic sequence analysis revealed that *P. pectinifera* GSS is a member of the relaxin‐type peptide family. Therefore, the GSS identified in *P. pectinifera* has been designated as relaxin‐like gonad‐stimulating peptide or RGP (Haraguchi et al., 2016). Subsequently, orthologs of *P. pectinifera* RGP (PpeRGP) have been identified in other starfish species, including *Asterias amurensis, Asterias rubens*, and *Aphelasterias japonica* (Mita et al., 2015; Mita and Katayama, 2016; Semmens et al., 2016).

The hormone relaxin was first discovered based on the observation that injection of serum from pregnant guinea pigs or rabbits caused relaxation of the pubic ligament of virgin guinea pigs (Hisaw, 1926). The ovarian corpus luteum and other parts of the reproductive tract were identified as sources of relaxin and its physiological role as a hormonal regulator of processes associated with preparation for parturition in mammals was established (Sherwood, 2004). Determination of the structure of relaxin revealed that it is an insulin‐like dimeric peptide comprising an A and B chain that are linked by disulfide bridges (Schwabe and McDonald, 1977). Subsequently, it was discovered that the prototypical ovarian relaxin belongs to a family of relaxin/insulin‐like peptides, which also occur in nonmammalian vertebrates (Bathgate et al., 2002; Hsu et al., 2005). Furthermore, investigation of the expression and functions of these peptides has revealed roles that extend beyond reproductive physiology. For example, relaxin‐3 is a neuropeptide expressed by neurons located in the nucleus incertus of the brain stem that project to many other regions of the brain. Accordingly, relaxin‐3 regulates a variety of processes, including arousal, stress, feeding, metabolism, and memory (Smith et al., 2011).

Starfish RGP was the first relaxin‐type peptide to be functionally characterized in an invertebrate (Mita et al., 2009a). Furthermore, its role as a gonadotropic peptide in starfish is interesting because it suggests that the prepartum actions of relaxin in mammals may reflect an evolutionarily ancient role in regulation of reproductive processes. The discovery of RGP has enabled investigation of its expression profile in *P. pectinifera*. Quantitative polymerase chain reaction (PCR) revealed expression in the radial nerve cords, consistent with the original discovery of GSS/RGP in this tissue. However, expression was also detected, albeit at much lower levels, in the cardiac stomach and tube feet (Mita et al., 2009a), which suggests that RGP may have nonreproductive functions in starfish. Analysis of RGP expression in *P. pectinifera* at the cellular level, using mRNA in situ hybridization techniques, has revealed that it is expressed by a population of cells located in the ectoneural epithelium of the radial nerve cords (Mita et al., 2009a). However, a wider analysis of RGP expression in the starfish body using mRNA in situ hybridization techniques has, as yet, not been conducted. This is of interest because it is not known if the radial nerve cord is the physiological source of RGP that triggers gamete maturation and release in starfish.

Recently, analysis of radial nerve cord transcriptome sequence data obtained from the common European starfish *A. rubens* enabled identification of a transcript encoding an RGP‐type precursor protein in this species (Semmens et al., 2016). Here we cloned and sequenced a cDNA encoding the *A. rubens* RGP (AruRGP) precursor, confirmed the presence of AruRGP in extracts of *A. rubens* radial nerve cords using mass spectrometry, and demonstrated the bioactivity of synthetic AruRGP in triggering oocyte maturation and ovulation. Furthermore, we used mRNA in situ hybridization to analyze the expression of AruRGP throughout the body of *A. rubens*, which has enabled visualization of cells expressing RGP in the radial nerve cords and in other parts of the body. Our findings provide important new insights into how RGP may mediate hormonal control of gamete maturation and release in starfish.

## MATERIALS AND METHODS

### Animals

Starfish (*A. rubens*) were collected at low tide from the Thanet coast (Kent, UK) or were obtained from a fisherman based at Whitstable (Kent, UK). The animals were maintained in a circulating seawater aquarium at ∼12°C in the School of Biological & Chemical Sciences at Queen Mary, University of London, and were fed on mussels (*Mytilus edulis*).

### cDNA cloning and sequencing and sequence analysis

BLAST analysis of *A. rubens* neural transcriptome sequence data using the PpeRGP precursor as a query identified a 2,915 base contig (1122961) encoding a 109 residue RGP‐type precursor protein (GenBank: KT601728) (Semmens et al., 2016). To confirm this contig sequence, which was obtained by assembly of Illumina HiSeq reads, and to obtain a template for probe synthesis, a cDNA encoding the *A. rubens* RGP (AruRGP) precursor was cloned and sequenced.

Total RNA was extracted from radial nerves of *A. rubens* using the SV Total RNA Isolation System (Promega, Madison, WI) and cDNA was synthesized using the QuantiTect Rev. Transcription Kit (Qiagen, Chatsworth, CA). A cDNA including the entire coding region of the AruRGP precursor transcript was amplified by PCR using Phusion high‐fidelity PCR master mix (New England Biolabs, Beverly, MA) with the oligonucleotide primers 5′‐ATGGCAAACTACCGTCTCAT‐3′ and 5′‐GCCACCCATGAAATAGTCAA‐3′ (custom synthesized by Sigma‐Aldrich, St. Louis, MO). The PCR product was gel‐extracted and purified using a QIAquick gel extraction kit (Qiagen). Then the AruRGP cDNA was cloned into pBluescript SKII (+) vector (Agilent Technologies, Palo Alto, CA), which was cut with the EcoRV‐HF restriction endonuclease (New England Biolabs) for sequencing.

The ExPASy translate tool (http://web.expasy.org/translate/) was used to determine the protein sequence of the AruRGP precursor and SignalP 4.1 (http://www.cbs.dtu.dk/services/SignalP/) was used to predict the signal peptide. Comparison of the sequence of the AruRGP precursor protein with RGP‐type precursors identified previously in other species (Mita et al., 2009a, 2015; Mita and Katayama, 2016) was performed using ClustalW (Thompson et al., 1994). Phylogenetic analysis of the relationship of AruRGP with other relaxin‐like peptides was investigated using the neighbor‐joining method (Zuckerkandl and Pauling, 1965; Felsenstein, 1985; Saitou and Nei, 1987) with MEGA 7.0.14 (Kumar et al., 2016).

### Preparation of extracts of *A. rubens* radial nerve cords for mass spectrometry

Extracts of *A. rubens* radial nerve cords were prepared to enable use of mass spectrometry to investigate the presence and structure of AruRGP in this tissue. Radial nerve cords were dissected from specimens of *A. rubens* as described previously (Chaet, 1964) and then transferred into 90% methanol / 9% acetic acid with or without the addition of protease inhibitors (pepstatin A [0.01 mM]; phenylmethylsulfonyl fluoride [PMSF; 0.1 mM]). The tissue was sonicated (two 2‐min pulses with 15‐sec intervals) and homogenized to lyse cells. The extract was centrifuged (10,000*g* for 5 min at 4°C) and the supernatant transferred to a glass vial. Finally, the solvent was bubbled off using nitrogen gas before being stored at –20°C.

Aliquots of 10 μl of radial nerve extract were diluted using 50 μl of 1 mM ammonium bicarbonate (Sigma Aldrich) to neutralize the high concentration of acetic acid used for extraction. Some aliquots were also subject to reduction to break disulfide bridges followed by alkylation of cysteine residues. For reduction, samples were treated with 5 μl of 100 mM dithiothreritol (Sigma Aldrich) and heated at 60°C for 15 minutes. Alkylation was performed by adding 5 μl of 200 mM iodoacetamide (Sigma Aldrich) and incubated in the dark at room temperature for 30 minutes. Samples of both reduced/alkylated and nonreduced/nonalkylated material were also digested using 0.5 μg trypsin (Promega) solution and incubated overnight at 37°C, with the digest arrested by addition of 10 μl of 10% formic acid (J.T. Baker, Phillipsburg, NJ).

### Mass spectrometry

NanoLC‐ESI‐MS/MS was used to analyze samples of radial nerve extracts, with a 3 μl aliquot of each sample separated by reversed phase chromatography prior to mass spectrometric analysis. Two columns were utilized, an Acclaim PepMap μ‐pre‐column cartridge (300 μm i.d. × 5 mm 5 μm 100 Å) and an Acclaim PepMap RSLC (75 μm × 25 cm 2 μm 100 Å) (Thermo Scientific), installed on an Ultimate 3000 RSLCnano system (Dionex, Sunnyvale, CA). Mobile phase buffer A was 0.1% formic acid in water and mobile phase B was 0.1% formic acid in acetonitrile. Samples were loaded onto the μ‐pre‐column equilibrated in 2% aqueous acetonitrile containing 0.1% trifluoroacetic acid for 8 minutes at 10 μl min^−1^, after which peptides were eluted onto the analytical column at 300 nl min^−1^ by increasing the mobile phase B concentration from 4% B to 25% over 90 minutes then to 35% B over 10 minutes and 90% B over 5 minutes, followed by a 15‐minute re‐equilibration at 4% B. Peptides were injected directly from the LC (300 nl min^−1^) via a Triversa Nanomate nanospray source (Advion Biosciences, NY) into a Thermo Orbitrap Fusion (Q‐OT‐qIT, Thermo Scientific, Pittsburgh, PA) mass spectrometer. Survey scans of peptide precursors from 400 to 1600 *m/z* were performed at 120K resolution (at 200 *m/z*) with automatic gain control (AGC) 5 × 10^5^. Precursor ions with charge state 2–6 were isolated (isolation at 1.2 Th in the quadrupole) and subjected to HCD fragmentation with normalized collision energy of 35. Tandem mass spectrometry (MS/MS) data were analyzed using the Orbitrap at 30K resolution, AGC was set to 5.4 × 10^4^, and the max injection time was 200 ms. Dynamic exclusion duration was set to 60 seconds with a 10 ppm tolerance around the selected precursor and its isotopes. Monoisotopic precursor selection was turned on. The instrument was run in top speed mode with 2‐second cycles.

### Analysis of mass spectrometry data

Raw data were converted to mascot generic format using MSConvert in ProteoWizard Toolkit (v. 3.0.5759) (Kessner et al., 2008). MS spectra were searched with Mascot engine (Matrix Science, v. 2.4.1) (Nesvizhskii et al., 2003) against a database comprising 40 *A. rubens* neuropeptide precursor proteins (Semmens et al., 2016), all proteins in GenBank from species belonging to the family Asteriidae and the common Repository of Adventitious Proteins Database (http://www.thegpm.org/cRAP/index.html). Theoretical peptides were generated from a tryptic digestion allowing up to two missed cleavages and variable modifications; carbamidomethyl on cysteine, oxidation on methionine, amidation (by modification of C‐terminal glycines), and pyroglutamate (by modification of N‐terminal glutamines). A no‐enzyme search was performed for samples not treated with trypsin. Precursor mass tolerance was 10 ppm and product ions were searched at 0.05 Da tolerances. Scaffold (v. Scaffold_4.6.1, Proteome Software) was used to validate MS/MS‐based peptide and protein identifications. Peptide identifications were accepted if they could be established at greater than 95.0% probability by the Scaffold Local FDR algorithm. Protein identifications were accepted if they could be established at greater than 95.0% probability and contained at least two identified peptides. Protein probabilities were assigned by the Protein Prophet algorithm (Nesvizhskii et al., 2003). Proteins that contained similar peptides and could not be differentiated based on MS/MS analysis alone were grouped to satisfy the principles of parsimony. Proteins sharing significant peptide evidence were grouped into clusters. The program Stavrox (v. 3.6.0) was used to search for crosslinked spectra (Gotze et al., 2012). Default settings were used except that semitryptic digestion was permitted, disulfide bonds were specified as the crosslinker, and high mass accuracy data were used with precursor mass tolerance of 5 ppm and fragment mass tolerance of 15 ppm. The full‐length AruRGP precursor was used as the reference sequence.

### Comparison of the effects of synthetic AruRGP and PpeRGP on *A. rubens* ovarian fragments

*Asterias rubens* RGP (AruRGP) and *P. pectinifera* RGP (PpeRGP) were synthesized commercially by the Peptide Institute (Osaka, Japan) and their bioactivity was assayed using ovarian fragments from *A. rubens*, as described previously (Shirai, 1986). Modified van't Hoff's artificial seawater (ASW) adjusted to pH 8.2 with 0.02 M borate buffer was prepared (Kanatani and Shirai, 1970) and the ovaries of mature female starfish were excised and cut using scissors into small fragments containing only a few lobes. The ovarian fragments were then incubated in ASW containing synthetic AruRGP or PpeRGP at a range of concentrations (5 × 10^−8^ to 4 × 10^−10^ M) for 1 hour. The samples were examined to determine whether or not spawning had occurred and were scored (Shirai, 1986) as follows: (+++) spawning occurred and most of the oocytes were matured; (++) about 50% of the oocytes were matured; (+) a few oocytes were matured; and (–) no spawning occurred. The scores were converted to numerical values (+++ = 100; + + = 67; + = 33; − = 0) so that the median effective concentration (EC_50_) could be determined graphically. Means ± SEM were determined from five separate assays using ovaries from different animals.

### Synthesis of digoxygenin‐labeled RNA probes for AruRGP precursor transcripts

A pBluescript SKII (+) vector containing the cloned and sequenced AruRGP precursor cDNA was used to synthesize RNA probes. First, a routine PCR was performed using *Taq* DNA polymerase (*Taq* DNA Polymerase with Thermopol Buffer, New England Biolabs) and standard M13 primers (Forward: 5′‐GTAAAACGACGGCCAGTG‐3′, Reverse: 5′‐GGAAACAGCTATGACCATG‐3′, custom‐synthesized by Sigma‐Aldrich) to linearize the plasmid and amplify the insert. The PCR product, which included the AruRGP precursor cDNA sequence and T3 and T7 RNA polymerase sites, was purified using a QIAquick gel extraction kit (Qiagen).

RNA probes were synthesized from the PCR product using a digoxygenin (DIG)‐labeled nucleotide triphosphate mix (Roche, Nutley, NJ) supplemented with dithiothreitol (Promega), a placental RNase inhibitor (Promega), and RNA polymerases (New England Biolabs), according to the manufacturer's instructions. T3 or T7 RNA polymerase was used for synthesis of the antisense or sense probes, respectively. Reaction products were digested with RNase free DNase (New England Biolabs) to remove template DNA and then stored at –20°C in 25% formamide made up in 2× saline‐sodium citrate (SSC) buffer.

### Fixation and sectioning of starfish

Specimens of *A. rubens* (diameter 4–6 cm) were fixed in 4% paraformaldehyde (PFA) in phosphate‐buffered saline (PBS, pH 7.4) overnight at 4°C. Different protocols were used to prepare paraffin‐embedded sections or frozen sections of fixed tissue.

To prepare specimens for embedding in paraffin wax, fixed starfish were cut with scissors to separate the five arms from the central disk region. Tissues were washed in autoclaved PBS for 10 minutes and then transferred to Morse's solution (10% sodium citrate; 20% formic acid in autoclaved water) for decalcification (typically 3 hours for arms and 8 hours for central disk). Then tissues were washed in distilled water for 10 minutes and dehydrated through a graded series of ethanol (50%, 70%, 90%, 3 × 100%; 30 minutes for each step). After clearing in xylene (1 × 5 minutes and 1 × 8 minutes; VWR Chemicals, Chicago, IL), the tissue was incubated in molten paraffin wax (3 × 1 hour) in an oven at ∼58°C. The tissue was embedded in wax using L‐shaped brass molds and stored at room temperature. Sections (12 μm) were cut using a Leica RM2145 microtome and collected on poly‐L‐lysine‐coated slides (Polysine; VWR).

To enable visualization of the pigmented optic cushion located at the tips of the starfish arms, frozen sections of arm tips were also prepared because the pigment is lost with the wax‐embedding method. After fixation, arm tips were washed in PBS (3 × 5 minutes) and then cryoprotected by incubation in sucrose solutions of ascending concentration (10%, 20%, 30% sucrose for 3 hours each step, at room temperature). The arm tips were embedded in RA Lamb OCT embedding cryoembedding Matrix (Fischer Scientific) and immediately frozen on dry ice. Sections (12 μm) were cut using a Leica CM3050 S cryomicrotome and collected on poly‐L‐lysine‐coated slides (Polysine; VWR) and then stored at –20°C.

### Localization of AruRGP transcripts using mRNA in situ hybridization

To increase adherence of sections, slides with paraffin wax‐embedded sections were placed in an oven at 65°C for 45 minutes followed by 15 minutes at room temperature to cool down. Slides were then incubated in xylene (3 × 7 min; VWR Chemicals) to remove the wax and rehydrated through a descending ethanol series (2 × 7 minutes 100%, 1 × 7 minutes 90%, 1 × 7 minutes 70%, 1 × 7 minutes 50%, 1 × 7 minutes 30%). The slides were then washed in PBS (2 × 7 minutes) and postfixed in 4% PFA/PBS for 20 minutes at room temperature. Following washes in PBS/0.1% Tween‐20 (National Diagnostics, Manville, NJ) (PBST; 3 × 5 minutes), sections were incubated at 37°C for 12 minutes in Proteinase K (Qiagen) solution at a concentration of 10 μg/ml in a buffer containing 50 mM Tris‐HCl (pH 7.5) and 6.25 mM EDTA. Sections were postfixed in 4% PFA/PBS for 5 minutes at room temperature and washed in PBST (3 × 5 minutes). Sections were then acetylated for 10 minutes in 1.325% triethanolamine (pH 7–8) (VWR Chemicals), 0.25% acetic anhydride (VWR Chemicals), and 0.175% acetic acid (VWR Chemicals) made up in distilled water, with continuous stirring. After washes in PBST (2 × 5 minutes) and 5× SSC (5 minutes) at room temperature, slides were prehybridized in hybridization buffer (50% formamide (Amresco, Solon, OH); 5× SSC; 500 μg/ml yeast total RNA (Sigma‐Aldrich); 50 μg/ml heparin (Sigma‐Aldrich); 0.1% Tween‐20 in distilled water) in a humid chamber for 2 hours. Hybridization was performed overnight in a humid chamber at 65°C by incubation of slides in hybridization buffer containing 800 ng/ml of denatured DIG‐labeled mRNA antisense or sense probes (100 μl per slide) with a coverslip made of Parafilm (Bemis, Terre Haute, IN).

The following day the slides were incubated in 5× SSC with slight shaking until the parafilm coverslip had floated off. Then slides were washed in 0.2× SSC (2 × 40 minutes at 65°C followed by 10 minutes at room temperature) followed by buffer B1 (10 mM Tris‐HCl, pH 7.5; 150 mM NaCl in autoclaved water) for 10 minutes at room temperature. After blocking slides with 5% goat serum (Sigma‐Aldrich)/B1 buffer in a humid chamber for 2 hours at room temperature, slides were incubated with alkaline phosphatase (AP)‐conjugated anti‐DIG antibody (Roche) at 1:3,000 dilution in 2.5% goat serum/B1 buffer in a humid chamber overnight at 4°C.

On the third day, slides were washed in B1 buffer (3 × 5 minutes at room temperature) followed by buffer B3 (100 mM Tris‐HCl, pH 9.5; 100 mM NaCl; 50 mM MgCl_2_ in distilled water) for 10 minutes at room temperature. AP substrate was prepared in buffer B3 by adding 4.5 μl/ml of nitro‐blue tetrazolium chloride (NBT) (Amresco) stock solution (75 mg/ml) in 70% dimethylformamide (Avantor, formerly Mallinkrodt Baker, St. Louis, MO) and 3.5 μl/ml of 5‐bromo‐4‐chloro‐3′‐indolyphosphate p‐toluidine (BCIP) (Panreac AppliChem) stock solution (50 mg/ml in 70% dimethylformamide) and applied to slides (500 μl per slide). Slides were then incubated in a humid chamber and checked regularly. Once strong staining was observed, the reaction was terminated by washing slides in distilled water (3 × 5 minutes). Slides were then dried on a hotplate and then incubated in 100% ethanol (2 × 10 sec) followed by clearing in Histo‐Clear (National Diagnostics) for 2 × 7 minutes. Finally, slides were mounted with HistoMount (National Diagnostics) and coverslipped.

The method used for mRNA in situ hybridization on sections of frozen tissue was the same as described above but with the following modifications. The initial oven‐drying, dewaxing, and hydration steps were not necessary, so these steps were omitted and instead slides were dried at room temperature prior to washing in PBS (2 × 7 minutes). Following staining of sections, slides were mounted with an aqueous mounting medium (Hydromount; National Diagnostics) and coverslipped. Mounted slides were left at room temperature to dry for 1–2 hours and then stored at 4°C.

Photographs of sections were captured with a QIClick CCD Camera (01‐QICLICK‐R‐F‐CLR‐12; QImaging) linked to a DMRA2 light microscope (Leica), using Volocity v.6.3.1 image analysis software (Perkin‐Elmer, Boston, MA) running on an iMac computer (27‐inch with OS X Yosemite, v. 10.10). Images were compiled into montages and labeled using Photoshop CC (2015.0.0; Adobe Systems, San Jose, CA), including use of cropping and contrast adjustment tools, running on a MacBook Pro computer (13‐inch, with OS X EI Capitan).

### Immunohistochemistry using monoclonal antibody 1E11

To facilitate interpretation of the expression pattern of AruRGP transcripts in starfish arm tips revealed using mRNA in situ hybridization methods, adjacent frozen sections were processed for immunohistochemical analysis using monoclonal antibody 1E11, which was generously provided by Dr. Robert D. Burke (University of Victoria, Canada; RRID AB_2617214). 1E11 is a neuron‐specific antibody to synaptotagmin B and is a marker of neural structures in echinoderms, including starfish (Burke et al., 2006; Saha et al., 2006). Importantly, the specificity of 1E11 for synaptotagmin B has been demonstrated by western blot analysis of radial nerve extracts from the sea urchin *Strongylocentrotus purpuratus* and comparison of immunostaining patterns observed with 1E11 and with antibodies to *S. purpuratus* synaptotagmin B. Further evidence of the specificity of 1E11 has been obtained by comparison of the distribution of 1E11 immunoreactivity with the distribution of synaptotagmin B mRNA in sea urchin revealed using mRNA in situ hybridization methods (Burke et al., 2006).

For immunohistochemistry with monoclonal antibody 1E11, starfish arm tips were lightly fixed (up to 30 minutes in 4% PFA/PBS; pH 7.4) because immunostaining with the 1E11 antibody is fixation‐sensitive (R.D. Burke, pers. commun.). Frozen sections of starfish arm tips mounted on slides were washed in PBS and then incubated for 20 minutes in PBS containing 1% hydrogen peroxide to quench endogenous peroxidases. Following washing with PBST, slides were blocked with 5% goat serum/PBST for 2 hours at room temperature. The slides were then incubated overnight at 4°C with the 1E11 antibody, diluted 1:3 with 5% goat serum/PBST. After washing with PBST, slides were then incubated for 3 hours at room temperature with goat antimouse horseradish peroxidase conjugated secondary antibodies immunoglobulins (Jackson ImmunoResearch, West Grove, PA) diluted 1:500 in PBST containing 2% goat serum. After washing in PBST, staining buffer (0.05% diaminobenzidine, 0.05% nickel chloride, 0.015% hydrogen peroxide in PBS) was applied to each slide until staining was observed. Slides were washed sequentially in PBS and autoclaved water and then coverslips were mounted using Hydromount (Natural Diagnostics). Photographs of immunostained sections and adjacent sections processed for AruRGP mRNA in situ hybridization were obtained as described above.

## RESULTS

### Cloning and sequencing of a cDNA encoding the *A. rubens* RGP precursor

**Figure 1:**
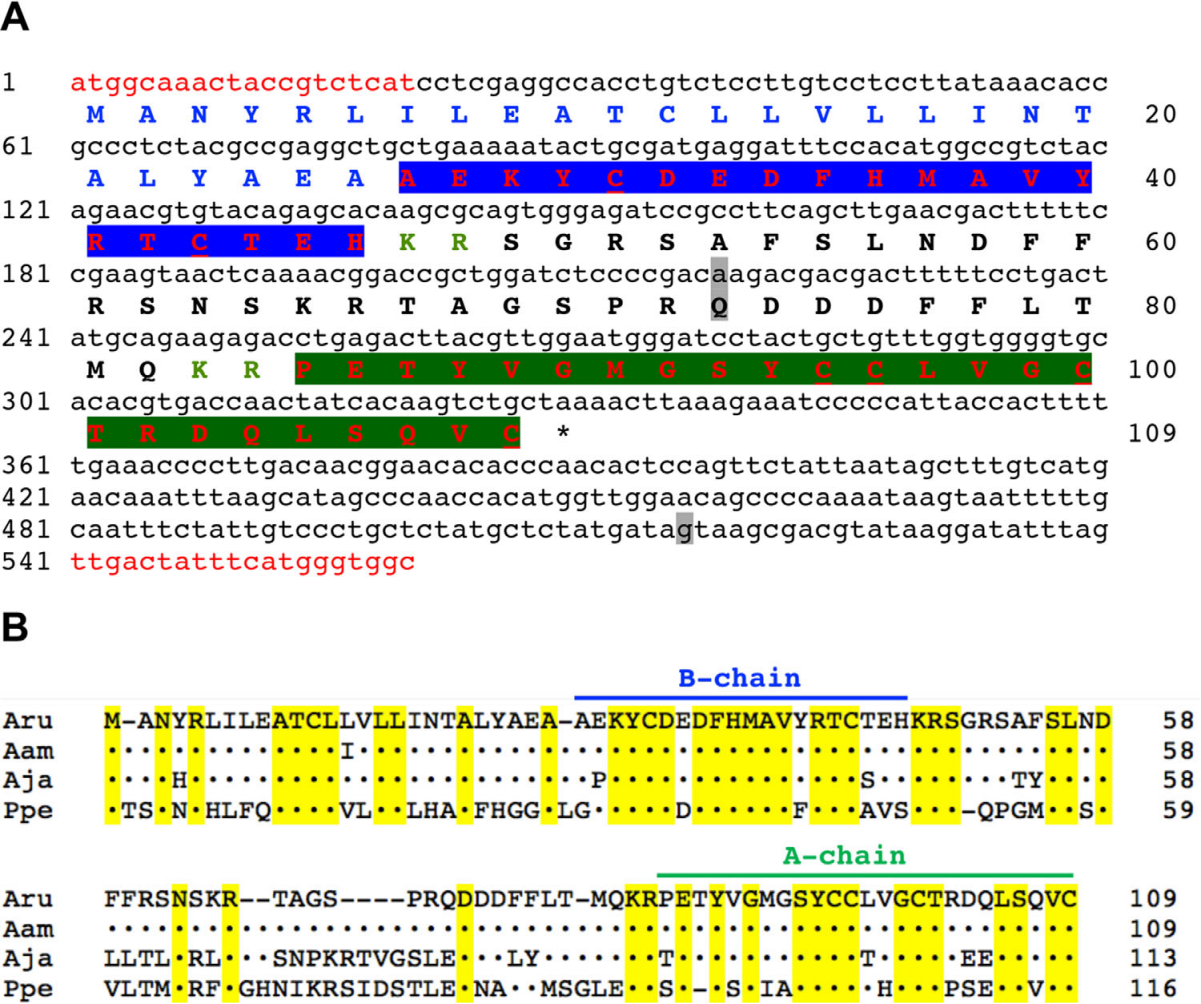
*A. rubens* relaxin‐like gonad‐stimulating peptide (AruRGP) precursor and comparison with RGP precursors from other starfish species. **A**: The cDNA sequence (lowercase, 560 bases) encoding the AruRGP precursor protein (uppercase, 109 amino acid residues) is shown. The predicted signal peptide is shown in blue and predicted dibasic cleavage sites are shown in green. Relaxin‐type peptides (with cysteine [C] residues underlined) are shown in red, with the A and B chains highlighted in green and blue, respectively. Nucleotides and amino acids that differ from the previously reported AruRGP precursor cDNA and protein sequence that was assembled from transcriptome sequence data (GenBank accession number KT601728; Semmens et al., 2016) are highlighted in gray. Nucleotide sequences that were used as primers for cDNA cloning are shown in red and the asterisk shows the position of the stop codon. **B**: Alignment of the AruRGP precursor with RGP precursors from *A. amurensis* (AamRGP, GenBank accession number LC040882), *A. japonica* (AjaRGP, GenBank accession number LC104980), and *P. pectinifera* (PpeRGP, GenBank accession number AB496611). Regions of the precursors corresponding to the A and B chains are labeled (green and blue, respectively) and amino acid residues that are identical in all four precursors are highlighted in yellow.

A 560‐base cDNA encoding the AruRGP precursor was cloned and sequenced, which is shown in Figure 1A together with the encoded 109 amino acid AruRGP precursor sequence. The AruRGP precursor has a predicted 26 amino acid signal peptide followed by the B chain (20 amino acids) (Fig. 1A). The A chain (25 amino acids) is located at the C terminus of the precursor and the intermediate 38 amino acid C peptide, which includes three putative dibasic proteolytic sites (KR; Fig. 1A), is sandwiched between the A and B chains.

An alignment of the amino acid sequence of the AruRGP precursor with the sequences of RGP precursors from *A. amurensis* (AamRGP), *A. japonica* (AjaRGP), and *P. pectinifera* (PpeRGP) (Mita et al., 2009a, 2015; Mita and Katayama, 2016) is shown in Figure 1B. The amino acid sequence of the AruRGP precursor is very similar to that of the AamRGP precursor (99% identity) but it shares lower levels of similarity with the AjaRGP precursor (75% identity) and the PpeRGP precursor (45% identity) (Fig. 1B; Table 1). More specifically, comparison of the amino acid sequences of the A and B chains that form RGP reveals that AruRGP is identical to AamRGP. In contrast, the A and B chain of AruRGP share 84% and 90% identity, respectively, with the A and B chains of AjaRGP, and 53% and 73% identity, respectively, with the A and B chains of PpeRGP (Table 1).

**Table 1:** Table showing the % amino acid identity that AruRGP and regions of the AruRGP precursor share with RGP and regions of RGP precursors from *A. amurensis, A. japonica* and *P. pectinifera*. The values in parentheses are number of amino acids

Species	% identity (number of amino acid residues)					
	RGP	RGP precursor				
		Signal peptide	A chain	B chain	C peptide	Total
*Asterias amurensis*	100 (45)	96 (26)	100 (25)	100 (20)	100 (38)	99 (109)
*Aphelasterias japonica*	87 (45)	96 (26)	84 (25)	90 (20)	43 (42)	75 (113)
*Patiria pectinifera*	65 (43)	38 (29)	58 (24)	73 (19)	23 (44)	45 (116)

**Figure 2:**
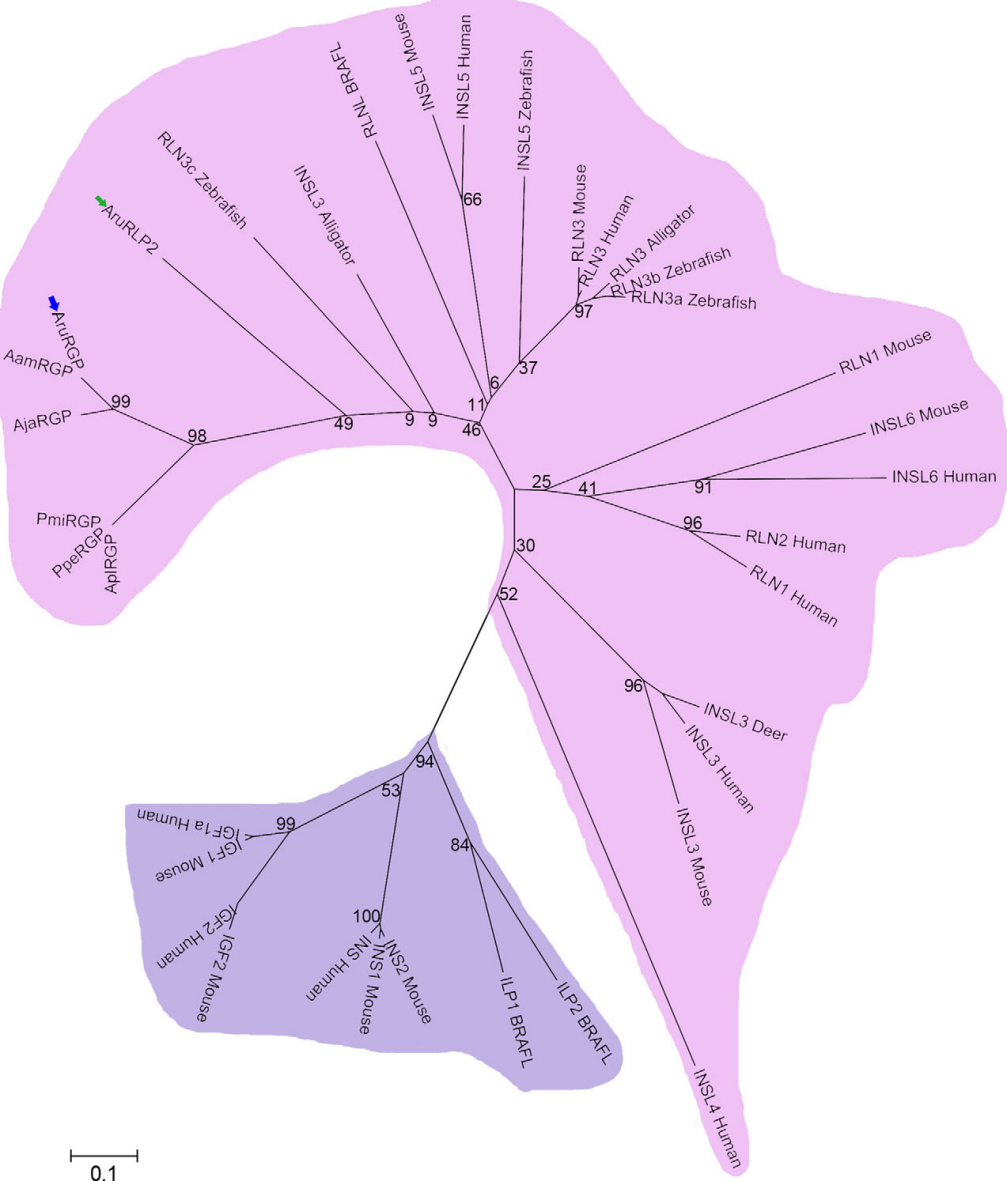
Neighbor joining tree showing the relationships of starfish relaxin‐like gonad‐stimulating peptide precursors with precursors of other members of the relaxin/insulin/insulin‐like growth factor (IGF) peptide family. The *A. rubens* RGP (AruRGP) precursor (blue arrow) and other starfish RGP precursors form a distinct clade within the relaxin/insulin‐like precursor family, which is highlighted in pink to distinguish it from the insulin/IGF precursor family that is highlighted in purple. A second *A. rubens* relaxin‐type precursor (AruRLP2; green arrow) is a paralog of the AruRGP precursor that is also positioned within the relaxin/insulin‐like clade of precursors. The full names and accession numbers of the 36 protein sequences included in the tree are as follows: AruRGP, relaxin‐like gonad‐stimulating peptide (ALJ99970.1, *Asterias rubens*); AamRGP, relaxin‐like gonad‐stimulating peptide precursor (BAR40315.1, *Asterias amurensis*); AjaRGP, relaxin‐like gonad‐stimulating peptide precursor (BAU20369.1, *Aphelasterias japonica*); PpeRGP, relaxin‐like gonad‐stimulating peptide precursor (BAI44654.1, *Patiria pectinifera*); AruRLP2, relaxin‐like peptide precursor 2 (ALJ99971.1, *Asterias rubens*); AplRGP, relaxin‐like gonad‐stimulating peptide precursor (LC033566.1, *Acanthaster planci*); PmiRGP, relaxin‐like gonad‐stimulating peptide precursor (LC057656.1, *Patiria miniata*); RLN1 Human, relaxin 1 precursor (NP_008842.1, *Homo sapiens*); RLN2 Human, relaxin 2 precursor (NP_604390.1, *Homo sapiens*); RLN3 Human, relaxin 3 precursor (NP_543140.1, *Homo sapiens*); RLN1 Mouse, relaxin 1 precursor (NP_035402.2, *Mus musculus*); RLN3 Mouse, relaxin 3 precursor (NP_775276.1, *Mus musculus*); RLN3 Alligator, relaxin 3 precursor (XP_006023546.1, *Alligator sinensis*); INSL3 Alligator, insulin‐like 3 (XP_006017481.1, *Alligator sinensis*); RLN3a Zebrafish, relaxin 3a precursor (NP_001032892.1, *Danio rerio*); RLN3b Zebrafish, relaxin 3b precursor (NP_001108535.1, *Danio rerio*); RLN3c Zebrafish, relaxin 3c precursor (NP_001108525.2, *Danio rerio*); INS Human, insulin precursor (NP_000198.1, *Homo sapiens*); INSL3 Human, insulin‐like peptide 3 precursor, (NP_005534.2, *Homo sapiens*); INSL4 Human, insulin‐like peptide 4 precursor (NP_002186.1, *Homo sapiens*); INSL5 Human, insulin‐like peptide 5 precursor (NP_005469.2, *Homo sapiens*); INSL6 Human, insulin‐like peptide 6 precursor (NP_009110.2, *Homo sapiens*); INSL3 Mouse, insulin‐like 3 precursor (NP_038592.3, *Mus musculus*); INSL5 Mouse, insulin‐like peptide precursor (NP_035961.1, *Mus musculus*); INSL6 Mouse, insulin‐like peptide precursor (NP_038782.1, *Mus musculus*); INSL5 Zebrafish, insulin‐like 5 precursor (NP_001122028.1, *Danio rerio*); INSL3 Deer, relaxin‐like peptide (AAR25542.1, *Capreolus capreolus*); IGF1a Human, insulin‐like growth factor 1 precursor (NP_000609.1, *Homo sapiens*); IGF2 Human, insulin‐like growth factor 2 precursor (NP_000603.1, *Homo sapiens*); IGF1 Mouse, insulin‐like growth factor 1 precursor (NP_001104745.1, *Mus musculus*); IGF2 Mouse, insulin‐like growth factor 2 precursor (NP_034644.2, *Mus musculus*); INS1 Mouse, insulin‐1 precursor (NP_032412.3, *Mus musculus*); INS2 Mouse, insulin‐2 precursor (NP_032413.1, *Mus musculus*); RLNL BRAFL, relaxin‐like peptide (EEA41967.1, *Branchiostoma floridae*); ILPl BRAFL, insulin‐like peptide 1 precursor ((Mita et al., 2009b), *Branchiostoma floridae*); ILP2 BRAFL, insulin‐like peptide 2 precursor ((Mita et al., 2009b), *Branchiostoma floridae*). Bootstrap values for selected nodes are shown.

Phylogenetic analysis of the relationship of the precursors of AruRGP and other starfish RGPs with related precursor proteins from vertebrates and invertebrates revealed that starfish RGPs cluster in a clade comprising precursors of relaxin/insulin‐like peptides, which is distinct from a clade comprising insulin/insulin‐like growth factor (IGF)‐type precursors (Fig. 2). Interestingly, a second relaxin‐type precursor (AruRLP2) that was recently identified in *A. rubens* (Semmens et al., 2016) is also positioned within the branch of relaxin/insulin‐like peptide precursors that contains the starfish RGP precursors (Fig. 2).

### Mass spectrometric detection of AruRGP in *A. rubens* radial nerve extracts

**Figure 3:**
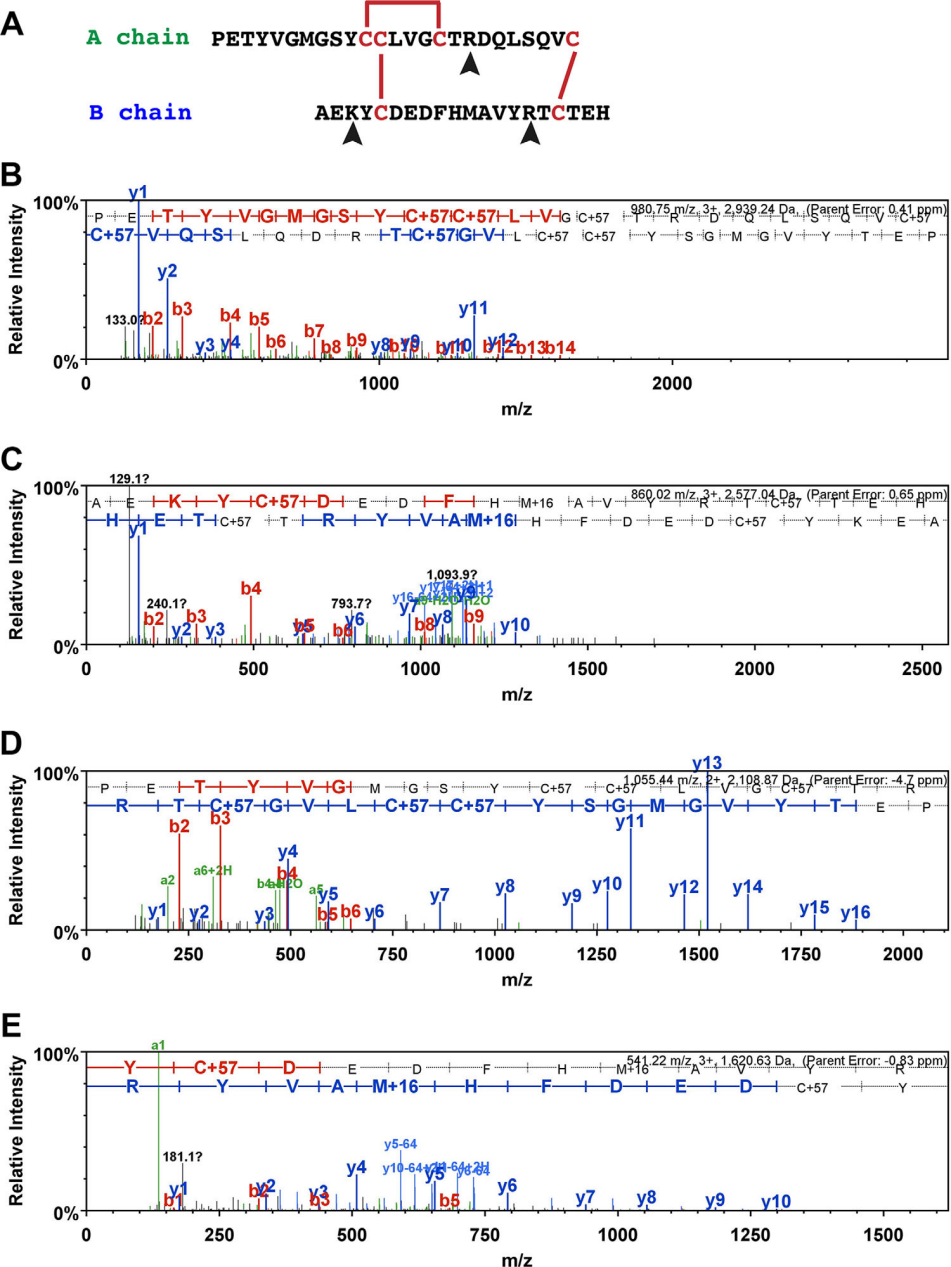
Mass spectrometric identification of AruRGP A chain and B chain in extracts of *A. rubens* radial nerve cords. **A**: Predicted dimeric structure of AruRGP, showing the sequences of the A chain and B chain. The positions of disulfide bridges are shown with red lines and tryptic cleavage sites are marked with arrowheads. **B,C**: MS/MS data for the A chain and B chain, respectively, from reduced and alkylated samples of radial nerve extract without tryptic digestion. The b series of peptide fragment ions are shown in red, the y series in blue and additional identified peptide fragment ions in green. The amino acid sequence identified in the mass spectrum is highlighted at the top of the figures. C+57 represents cysteine modified by carbamidomethylation and M+16 represents oxidized methionine. The observed *m/z* of the precursor ion for the A chain (PETYVGMGSYCCLVGCTRDQLSQVC; B) is 980.75 with a charge state 3 + and an error of 0.41 ppm between the experimentally determined and predicted values (Mascot score = 57). The observed *m/z* of the precursor ion for the B chain (AEKYCDEDFHMAVYRTCTEH; C) is 860.02 with a charge state of 3 + and an error of 0.65 ppm between the experimentally determined and predicted values (Mascot score = 31). **D,E**: MS/MS data for the complete sequences of fragments of the A chain and B chains, respectively, derived from reduced and alkylated samples of radial nerve extract subjected to tryptic digestion, with annotations in the same format as in B and C. The observed *m/z* of the precursor ion for the A chain fragment (PETYVGMGSYCCLVGCTR; D) is 1055.44 with a charge state of 2 + and an error of –4.7 ppm between the experimentally determined and predicted values (Mascot score = 98). The observed *m/z* of the precursor ion for the B chain fragment (YCDEDFHMAVYR; E) is 541.22 with a charge state of 3 + and an error of –0.83 ppm between the experimentally determined value and predicted value (Mascot score = 45).

**Figure 4:**
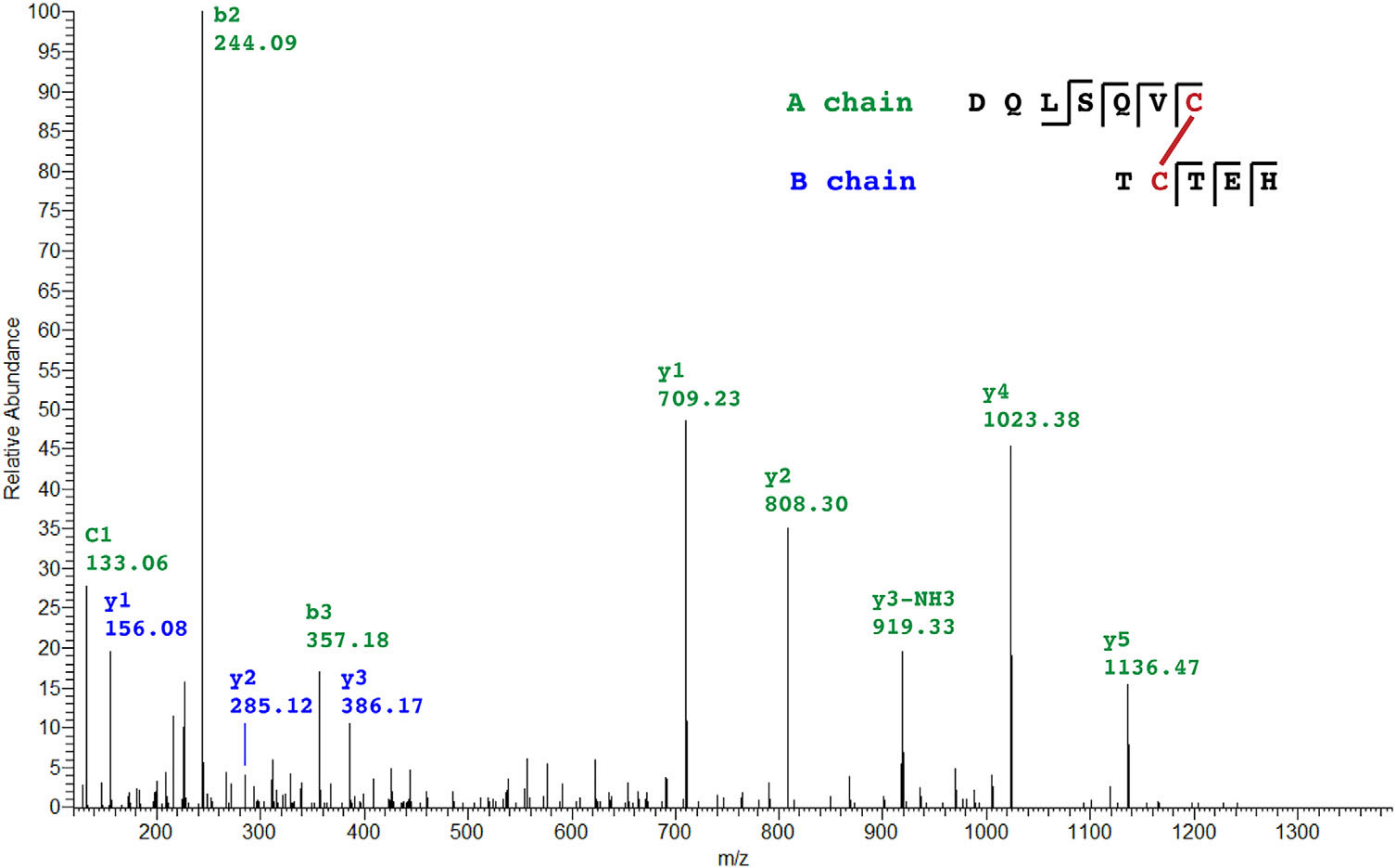
Mass spectrometric identification of a dimeric fragment of AruRGP in an extract of *A. rubens* radial nerve cords. The mass spectrum of a disulfide bridge linked dimeric peptide comprising DQLSQVC from the AruRGP A chain and TCTEH from the AruRGP B chain is shown. This dimeric peptide was detected in samples of radial nerve extract that were subjected to tryptic digestion without reduction. Peptide fragments from the A chain are shown in green and peptide fragments from the B chain are shown in blue. The observed *m/z* of the precursor ion is 690.28 with a charge state 2 + and an error of –0.73 ppm between the experimentally determined value and predicted value (Stavrox score = 145).

To determine if the AruRGP precursor (Fig. 1) is processed to form mature AruRGP in a manner consistent with other relaxin‐type peptides (Fig. 3A) (Schwabe and McDonald, 1977; Sherwood, 2004), we analyzed *A. rubens* radial nerve extracts with and without reduction/alkylation and with and without trypsin digestion. In samples subjected to reduction and alkylation, the A chain and B chain were identified by LC‐MS/MS, while in the absence of reduction these peptides were not detected, indicating that the A and B chains are linked by disulfide bridges. The A chain (PETYVGMGSYCCLVGCTRDQLSQVC) was observed as 3 + ions (980.75 *m/z*; Fig. 3B) and the B chain (AEKYCDEDFHMAVYRTCTEH) was observed as 3 + ions (860.02 *m/z;* Fig. 3C) and 4 + ions, with and without oxidized methionine. Although these long and highly charged peptides had significant Mascot scores (Fig. 3B,C), they fragmented poorly. Therefore, to obtain further confirmation of the sequences of A chain and B chain peptides we analyzed radial nerve extract samples incubated with trypsin, which cleaves after lysine and arginine residues to yield peptides that could be completely sequenced using MS/MS. Trypsin was used with or without prior reduction of the peptides and therefore in principle masses and spectra for both disulfide bridge crosslinked and unlinked peptides could be detected under the relevant conditions. In samples subjected to reduction followed by trypsin treatment, the expected fragments of the A chain (PETYVGMGSYCCLVGCTR [2 + 1055.44; Fig. 3D]) and B chain (YCDEDFHMAVYR [2 + 541.22; Fig. 3E]) were detected and sequenced, respectively.

Treatment of nonreduced samples of radial nerve extracts with trypsin would be expected, based on the predicted structure of AruRGP (Fig. 3A), to produce two dimeric peptides linked by single disulfide bridges. We were unable to detect the larger of these predicted peptides comprising the N‐terminal region of the A chain (PETYVGMGSYCCLVGCTR) linked to the central region of the B chain (YCDEDFHMAVYR). However, we were able to detect the smaller dimeric peptide comprising the C‐terminal regions of the A chain (DQLSQVC) and B chain (TCTEH) in two charge states (690.28, 2 + and 460.52 3+) (Fig. 4).

### Comparison of the effects of AruRGP and PpeRGP on ovarian fragments from *A. rubens*

**Figure 5:**
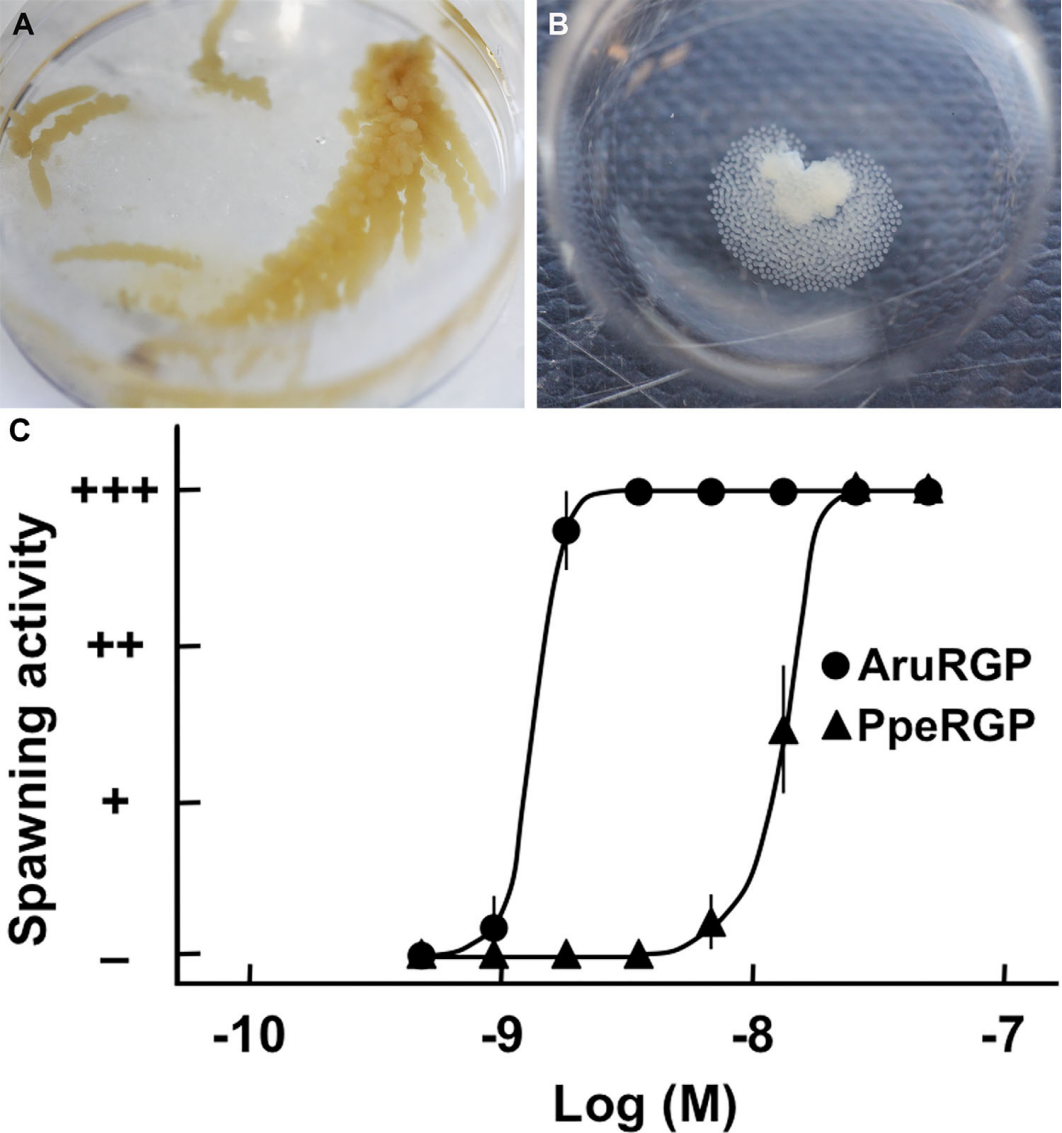
Comparison of the in vitro bioactivity of AruRGP and PpeRGP as inducers of spawning in *A. rubens*. **A**: Isolated ovary from *A. rubens*. **B**: AruRGP‐induced spawning of an ovary fragment from *A. rubens*. **C**: Graph showing the dose‐dependent effects of AruRGP (•) and PpeRGP (▴) in causing spawning of ovarian fragments. + + + denotes spawning occurred and most of oocytes were matured, + + denotes about 50% oocytes were matured, + denotes a few oocytes were matured, and – denotes no spawning occurred. Means ± SEM for five separate assays using ovarian tissue from different animals are shown. The median effective concentration (EC_50_) of AruRGP required to induce spawning (1.33 ± 0.09 nM) is approximately 10‐fold lower than for PpeRGP (14 ± 1 nM).

Previous studies have shown that AamRGP does not induce spawning of ovaries from *P. pectinifera*, whereas PpeRGP triggers spawning of ovaries from *A. amurensis* (Mita et al., 2015). Here the effects of AruRGP and PpeRGP on oocyte maturation and ovulation was examined in the isolated ovary of *A. rubens* (Fig. 5A). Synthetic AruRGP induced oocyte maturation and ovulation in ovarian fragments from *A. rubens* within 30 minutes (Fig. 5B). Both AruRGP and PpeRGP caused dose‐dependent induction of spawning of *A. rubens* ovarian fragments (Fig. 5C). However, the EC_50_ of AruRGP required to induce spawning (1.33 ± 0.09 nM) is approximately 10‐fold lower than for PpeRGP (14 ± 1 nM), consistent with differences in the sequences of AruRGP and PpeRGP (Fig. 1B; Table 1).

### Cellular localization of AruRGP precursor transcripts in *A. rubens*

**Figure 6:**
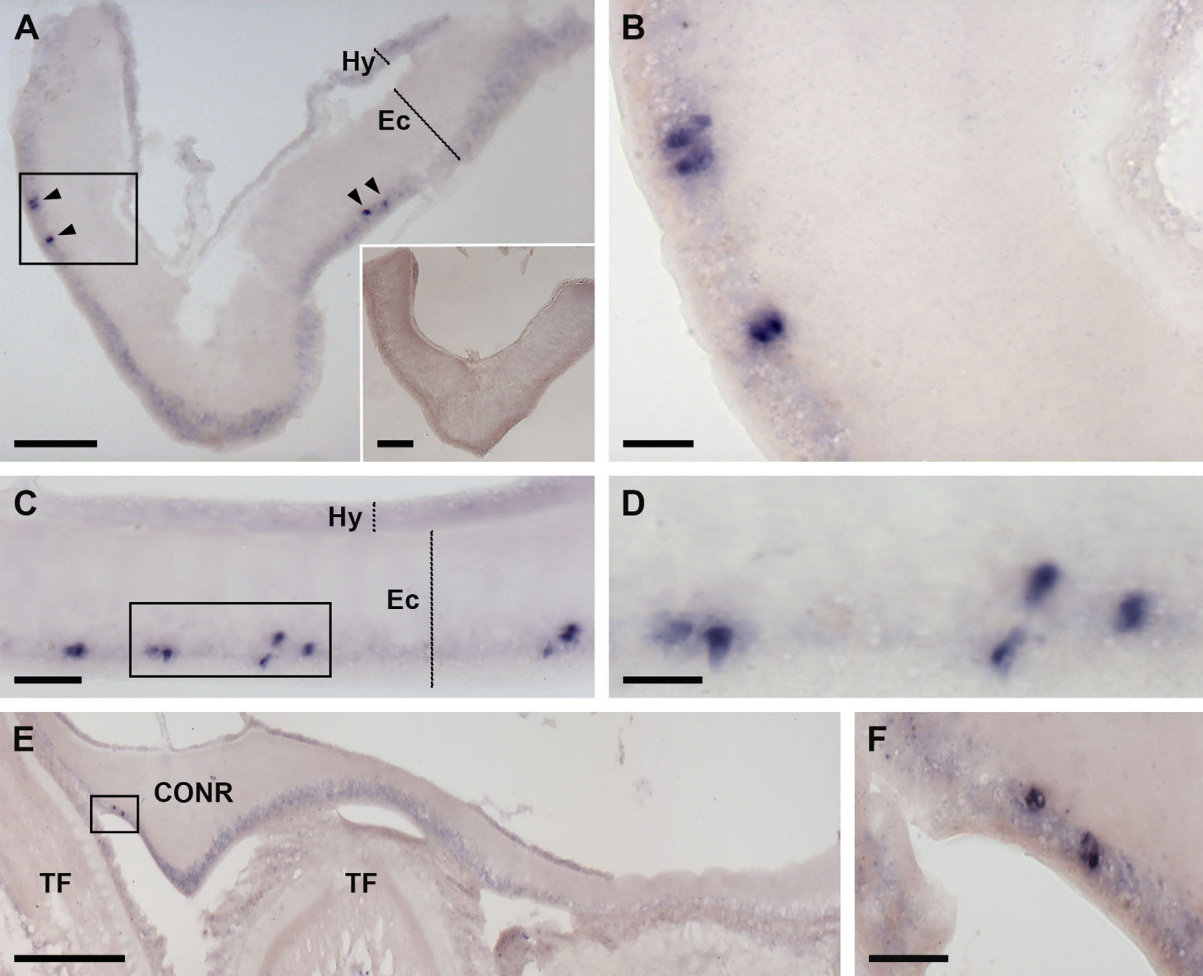
Localization of AruRGP precursor mRNA in the radial nerve cord and circumoral nerve ring of *A. rubens* using in situ hybridization. **A,B**: Transverse sections of radial nerve cord incubated with antisense probes (main panels of A and B) showing a bilaterally symmetrical group of 2–3 stained cells (arrowheads) in the epithelium of the ectoneural region of the nerve cord. Panel B shows a high‐magnification view of the rectangular region highlighted in panel A. The inset of panel A shows the absence of staining in a transverse section of radial nerve cord incubated with sense probes, demonstrating the specificity of staining observed with antisense probes. **C,D**: Longitudinal parasagittal sections of the radial nerve cords incubated with antisense probes showing groups of cells interspersed along the length of the nerve cord in the ectoneural epithelium. Panel D shows a high‐magnification view of the rectangular region highlighted in panel C. **E,F**: Transverse section of the disk region in *A. rubens* incubated with antisense probes, showing the circumoral nerve ring and tube feet. Stained cells can be seen in the ectoneural epithelium of the nerve cord, highlighted by the rectangle in E and shown at higher magnification in F. CONR, circumoral nerve ring; Ec, ectoneural region of radial nerve cord; Hy, hyponeural region of radial nerve cord; TF, tube foot. Scale bars = 50 μm in A and A inset; 10 μm in B,D; 25 μm in C; 200 μm in E; 20 μm in F.

**Figure 7:**
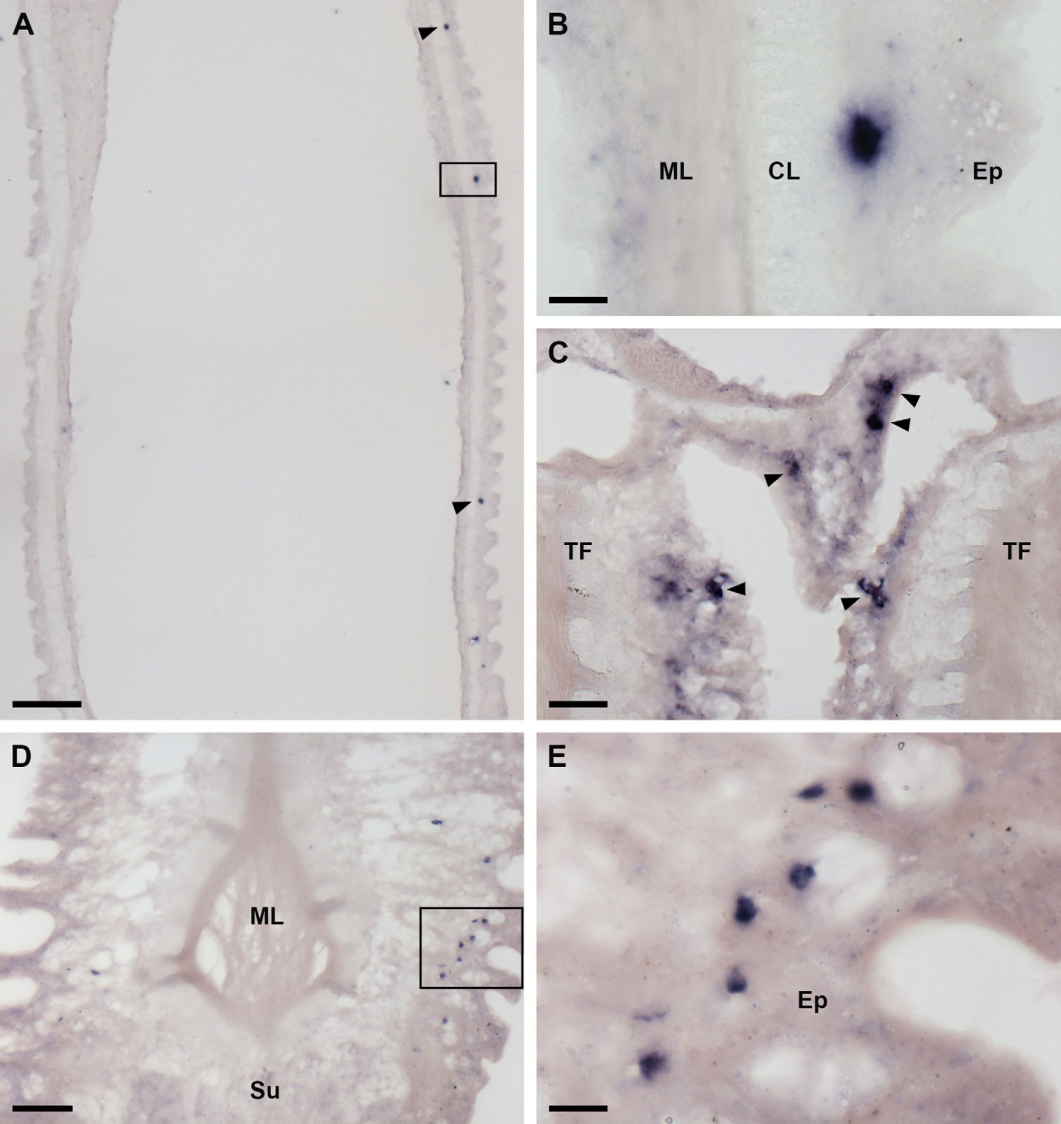
Localization of AruRGP precursor mRNA in tube feet of *A. rubens* using in situ hybridization. **A**: Longitudinal section of a tube foot showing three stained cells (arrowheads and rectangle) in the subepithelial layer of the podium. **B**: The region highlighted with a rectangle in A is shown here at higher magnification, with a stained cell located between the external epithelium and connective tissue layer. **C**: Stained cells (arrowheads) located in the subepithelial layer near to the base of adjacent tube feet. **D**: A group of stained cells (see rectangle) in the tube foot subepithelial layer just above the sucker. **E**: The region highlighted with a rectangle in D is shown here at higher magnification. CL, connective tissue layer; Ep, epithelium; ML, muscle layer; Su, sucker TF: tube foot. Scale bars = 100 μm in A; 10 μm in B; 25 μm in C; 50 μm in D; 10 μm in E.

Analysis of AruRGP precursor expression in *A. rubens* using mRNA in situ hybridization with antisense probes revealed stained cells in the radial nerve cords (Fig. 6A–D), in accordance with cloning of the AruRGP precursor cDNA from this tissue (see above) and previous studies on GSS/RGP in starfish. The specificity of this staining was confirmed by control experiments using sense probes, where no stained cells were observed (Fig. 6A, inset). The cells expressing AruRGP transcripts are located in the epithelium of the ectoneural region of the radial nerve cords (Fig. 6A–D). These cells are relatively few in number, with a bilaterally symmetrical group of two or three cells present on both sides of the V‐shaped radial nerve cord when viewed in transverse sections of the starfish arm (Fig. 6A,B). Analysis of longitudinal sections revealed that these groups of cells occur along the length of the radial nerve cord, separated by gaps of 20–80 μm (Fig. 6C,D). The radial nerve cords are linked by a circumoral nerve ring that is located in the central disk region of starfish (Pentreath and Cobb, 1972) and AruRGP‐expressing cells were also detected in the ectoneural epithelium of the nerve ring (Fig. 6E,F). The radial nerve cords and circumoral nerve ring control the coordinated activity of adjacent rows of tube feet located along the underside of each arm and around the mouth (Pentreath and Cobb, 1972). Cells expressing AruRGP transcripts were revealed in the tube feet in a subepithelial location along the length of the podium (Fig. 7A,B), at the base of the tube feet (Fig. 7C), and as a cluster of cells near to the tube foot sucker (Fig. 7D,E).

**Figure 8:**
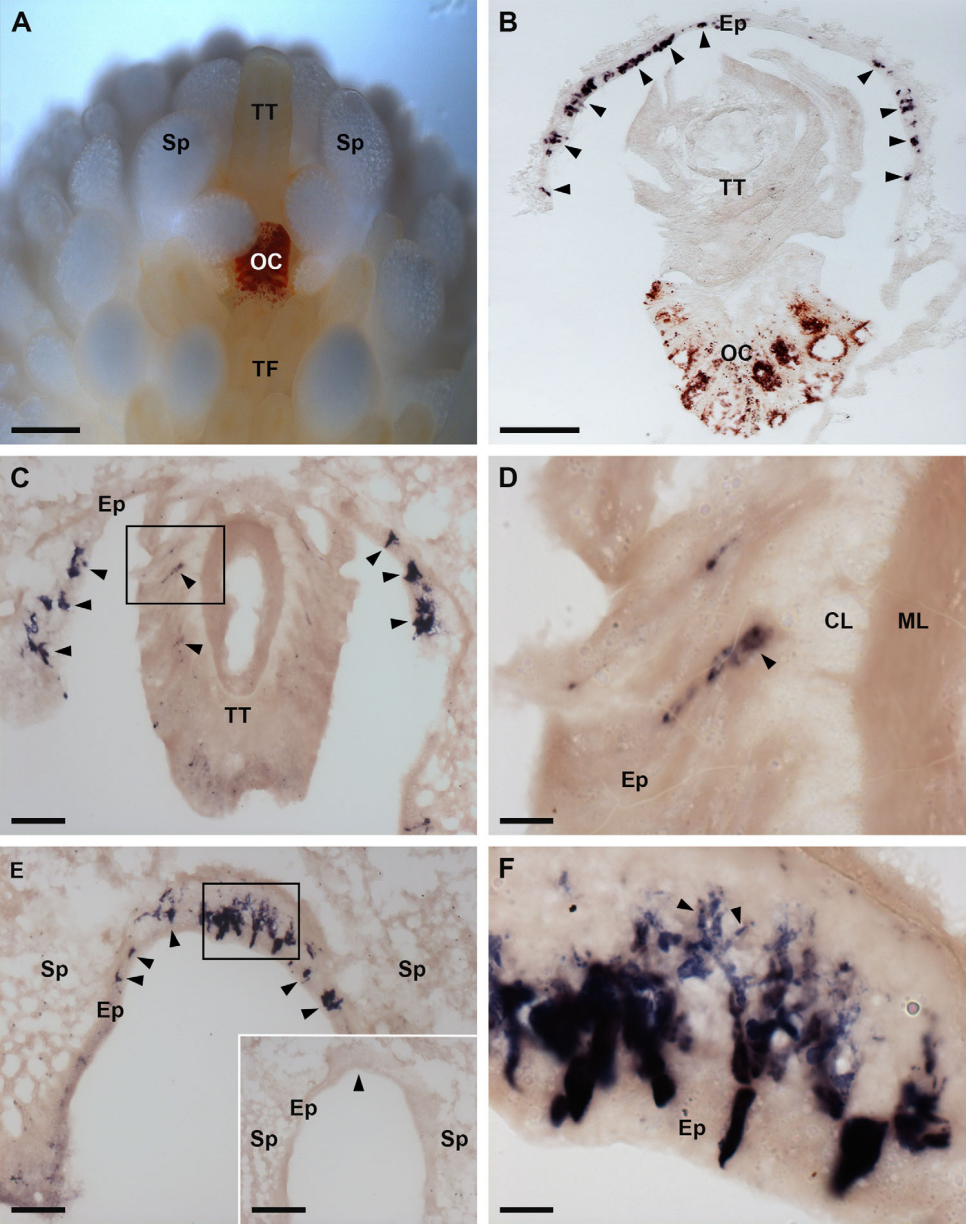
Localization of AruRGP precursor mRNA in the arm tips of *A. rubens* using in situ hybridization. **A**: Photograph of a living specimen of *A. rubens* showing the arm tip region viewed from the underside (oral) of the animal, taken using a Leica DFC420 C camera linked to a Leica S8 APO microscope. The most prominent feature is the pigmented optic cushion, which is located at the base of the terminal tentacle. The terminal tentacle and optic cushion are bounded on each side by spines and rows of tube feet can be seen adjacent to the optic cushion. **B**: Section of the arm tip showing the pigmented optic cushion and terminal tentacle. Stained cells expressing AruRGP precursor transcripts (arrowheads) can be seen in the body wall epithelium lining a cavity that surrounds the terminal tentacle and optic cushion. **C**: Section of an arm tip showing the terminal tentacle cut obliquely. Stained cells can be seen in the terminal tentacle (rectangle and arrowheads) and in the body wall epithelium at the base of the spines that surround the terminal tentacle (arrowheads). **D**: Detail of the region highlighted with a rectangle in panel C, showing stained cells (arrowhead) in the subepithelial layer of the terminal tentacle. **E**: Section through the distal region of the arm tip beyond the terminal tentacle, showing stained cells (arrowheads and rectangle) in the body wall epithelium at the base of two adjacent spines; the region highlighted with a rectangle is shown in panel F. The inset shows absence of staining (arrowhead) in a section of the arm tip adjacent to the section shown in the main panel and which was incubated with sense probes instead of the antisense probes used in the main panel E. **F**: Detail of the region highlighted with a rectangle in panel E, showing stained cells with processes (arrowheads) at high magnification. CL, connective tissue layer of terminal tentacle; Ep, epithelium of body wall; ML, muscle layer of terminal tentacle; OC, optic cushion; TF, tube foot; Sp, spine; TT, terminal tentacle. Scale bars = 400 μm in A; 100 μm in B, E inset; 50 μm in C,E; 10 μm in D,F.

The most striking expression of AruRGP transcripts was revealed in the tip regions of the arms (Fig. 8). To facilitate interpretation of the staining shown in Figure 8, a labeled photograph of the arm tip region of a live specimen of *A. rubens* is shown in Figure 8A. The most prominent feature of the arm tip is the pigmented optic cushion, which is located at base of a tube foot‐like organ specialized for sensory functions that is known as the terminal tentacle (Fig. 8A,B). Consistent with expression of AruRGP in tube feet (Fig. 7), cells expressing AruRGP transcripts were also revealed in the terminal tentacle (Fig. 8C,D). However, more extensive expression of AruRGP is present in the body wall epithelium that lines the cavity containing the terminal tentacle and optic cushion. Here AruRGP‐expressing cells are located in the epithelium forming the “ceiling” (Fig. 8B) and “walls” (Fig. 8C) of the cavity, extending right up to distal tip of the arm (Fig. 8E). The staining observed in these cells is not observed in sections of arm tips incubated with sense probes, demonstrating the specificity of the labeling (Fig. 8E, inset). Observation of clusters of AruRGP‐expressing cells in the arm tip body wall epithelium at high magnification revealed a meshwork of stained processes, suggesting that these cells are neurons (Fig. 8F).

**Figure 9:**
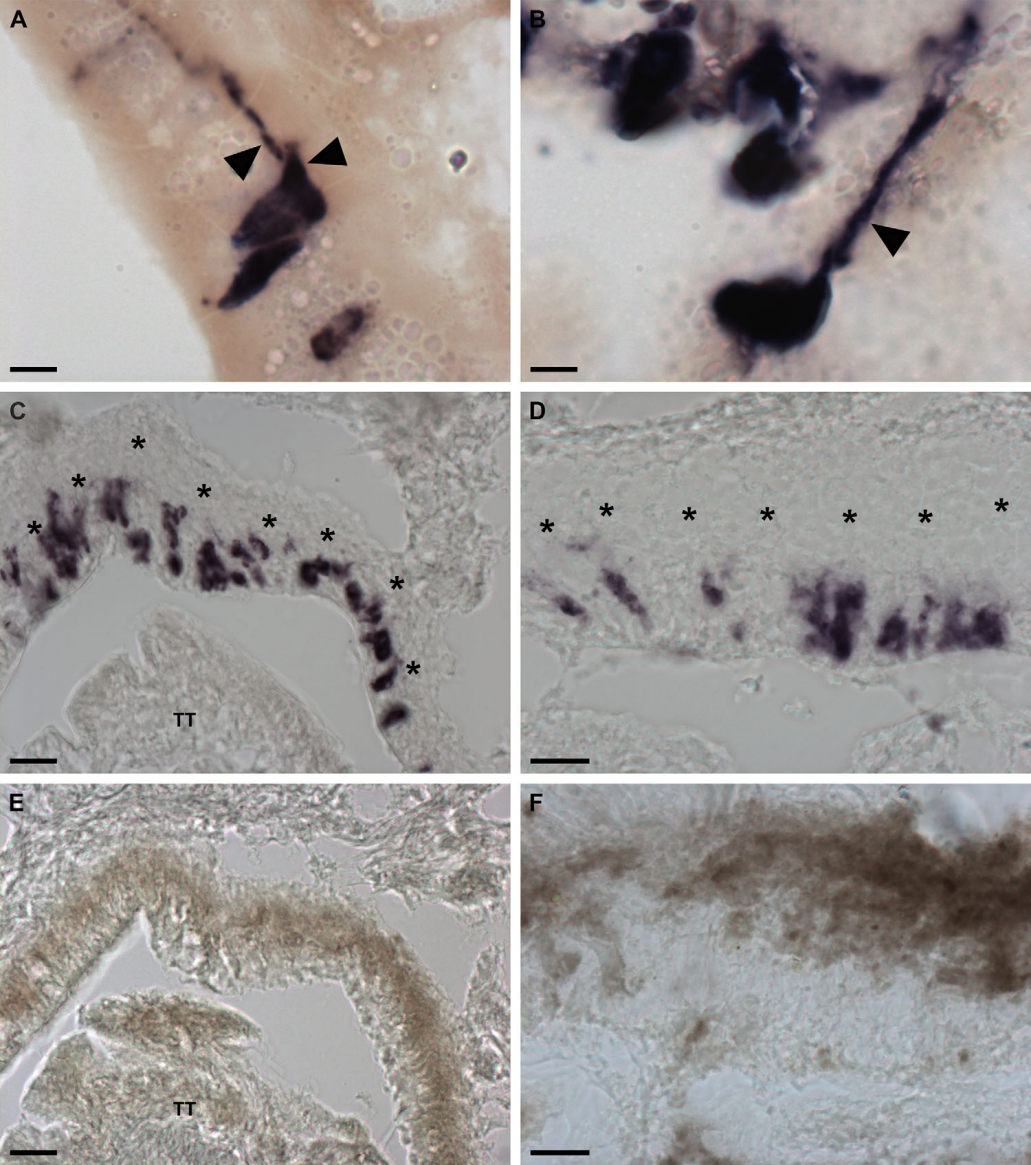
Neuron‐like characteristics of cells expressing AruRGP in the arm tips of *A. rubens*. **A**: Transverse section of *A. rubens* arm tip showing two cells in the body wall epithelium that express the AruRGP precursor, as revealed by mRNA in situ hybridization, and that have stained axon‐like processes (arrowheads). **B**: Longitudinal section of *A. rubens* arm tip showing a cell in the body wall epithelium that expresses the AruRGP precursor, as revealed by mRNA in situ hybridization, and that has a stained axon‐like process (arrowhead). **C,D**: Transverse sections of *A. rubens* arm tip showing cells expressing AruRGP precursor transcripts, as revealed by mRNA in situ hybridization, in the body wall epithelium lining the cavity that contains the terminal tentacle (TT). **E,F**: Transverse sections of *A. rubens* arm tip adjacent to the sections shown in panels C and D, respectively, showing that the unstained region in panels C and D underlying the AruRGP expressing cells (see asterisks in panels C and D) is immunoreactive with monoclonal antibodies (1E11) to the axonal protein synaptotagmin B. This provides supporting evidence that the AruRGP expressing cells in the arm tip epithelium are neurons. Scale bars = 5 μm in A,B; 20 μm in C,E; 10 μm in D,F.

To more specifically investigate if the AruRGP‐expressing cells in the arm tip body wall epithelium are neurons, both transverse and longitudinal sections of arm tips were analyzed at high magnification. This revealed examples of solitary cells or pairs of cells that clearly have stained processes emanating basally from an intraepithelial cell body (Fig. 9A,B). Furthermore, double‐labeling experiments using the neural‐specific antibody 1E11 revealed a layer of immunostained neural processes immediately beneath the layer of AruRGP‐expressing cell bodies in arm tip epithelium (Fig. 9C–F). Therefore, collectively these observations indicate that AruRGP‐expressing cells in the arm tip epithelium are neurons.

## DISCUSSION

We report here the identification and functional characterization of relaxin‐like gonad‐stimulating peptide in the starfish *A. rubens* (AruRGP). Analysis of the sequence of AruRGP revealed that it is similar to RGP from other starfish species. AruRGP is identical to RGP from another species in the genus *Asterias*—*A. amurensis*—but, as would be expected, AruRGP shares lower levels of similarity with RGP from more distant species. Thus, AruRGP shares 87% sequence identity with RGP from *Aphelasterias japonica*, which belongs to the same order and family as *Asterias* (Order Forcipulatida; Family Asteriidae), and only 65% sequence identity with RGP from *P. pectinifera* (PpeRGP), which belongs to a different order of starfish (Order Valvatida; Family Asterinidae). All of the starfish RGPs, including AruRGP, belong to a family of relaxin/insulin‐like peptides, which are distinct from insulin/IGF‐type peptides.

The dimeric structure PpeRGP comprising an A chain and B chain has been demonstrated previously (Mita et al., 2009a), with the positions of intrachain and interchain disulfide bridges inferred from vertebrate relaxins (Schwabe and McDonald, 1977; Sherwood, 2004). Accordingly, monomeric PpeRGP A chain or PpeRGP B chain when tested individually or as a mixture lack gonadotropic activity, whereas synthetic dimeric PpeRGP comprising the A and B chains with disulfide bridges in the same positions as in vertebrate relaxin exhibits gonadotropic activity that is indistinguishable from RGP purified from radial nerve extracts (Mita et al., 2009a). Here we used mass spectrometry to confirm the presence and sequences of both the A chain and B chain of AruRGP in *A. rubens* radial nerve extracts. Furthermore, we demonstrated the presence of one of the two disulfide bridges that link the A chain and B chain. Informed by these findings and the conserved structure of relaxin‐type peptides (Schwabe and McDonald, 1977; Sherwood, 2004), we had dimeric AruRGP synthesized with the structure shown in Figure 3A. The bioactivity of this synthetic AruRGP as a potent stimulator of spawning from *A. rubens* ovarian fragments was demonstrated here. Furthermore, as was found in a previous study on *A. amurensis* (Mita et al., 2015), AruRGP is 10‐fold more potent than PpeRGP, consistent with the differences in the sequences of AruRGP and PpeRGP.

The main novel objective of this study was to comprehensively analyze the expression of AruRGP in *A. rubens* using mRNA in situ hybridization. Consistent with the original discovery of GSS/RGP in extracts of radial nerve cords (Chaet and McConnaughy, 1959), cells expressing AruRGP transcripts were revealed in the epithelium of the ectoneural region of the radial nerve cords. These cells occur in bilaterally symmetrical groups of two or three cells along the length of the V‐shaped radial nerve cord separated by gaps. The relative abundance of these cells in the radial nerve cords of *A. rubens* is similar to findings from *P. pectinifera*, where bilaterally symmetrical groups of four or five stained cells were observed in transverse sections of the radial nerve cord (Mita et al., 2009a). However, the exact location of the RGP‐expressing cells in the radial nerve cord is different in *P. pectinifera* and *A. rubens*. In *P. pectinifera* the cells are located at the apex of the V‐shaped nerve cord, whereas in *A. rubens* the cells are located more laterally, about half way up the left and right sides of the V‐shaped nerve cord. The functional significance of these interspecies differences in the location RGP‐expressing cells in the radial nerve cord is not known. AruRGP‐expressing cells were also detected in the ectoneural epithelium of the circumoral nerve ring, which links the radial nerve cords in the central disk region of starfish.

Previous studies have revealed that GSS/RGP expression/activity is also detected in the cardiac stomach and tube feet of *P. pectinifera*, albeit at much lower levels than in the radial nerve cords (Mita et al., 2009a). Our analysis of AruRGP expression in *A. rubens* using mRNA in situ hybridization did not reveal expression in the cardiac stomach, which may reflect expression at levels below the threshold for detection with the methods used or an interspecies difference in the expression patterns of RGP. However, consistent with the detection of GSS/RGP in *P. pectinifera* tube feet (Mita et al., 2009a), we did reveal the expression of AruRGP by cells in the tube feet of *A. rubens*. Thus, groups of AruRGP‐expressing cells were observed at the base of the podia, interspersed along its length and concentrated in a cluster near to the tube foot sucker.

Because GSS/RGP was originally extracted from the radial nerve cords of starfish (Chaet and McConnaughy, 1959; Mita et al., 2009a), there has been an assumption that this tissue is the physiological source of GSS/RGP that then acts as a hormone to trigger gamete maturation and release (Chaet, 1966; Kanatani, 1979; Mita, 2013). Furthermore, prior to the identification of GSS as RGP, it was proposed that GSS is synthesized by supporting cells (Kanatani, 1979), which are radial glia‐like cells located in the epithelial layer of the ectoneural region of the nerve cord and that have processes (fibers) that extend across the ectoneural neuropile. It was postulated that secretory granules containing GSS are transported along the supporting cell fibers and their contents are released as hormones from the inner surface of the radial nerve cords (Unger, 1962). However, a difficulty with this hypothesis is that histochemical analysis of the radial nerve cords reveals that the supporting cell fibers terminate at the connective tissue boundary between the ectoneural and hyponeural regions of the radial nerve cords and do not reach as far as the inner surface (Mashanov et al., 2016). Furthermore, the number of cells expressing RGP in *P. pectinifera* (Mita et al., 2009a) and in *A. rubens* (this study) does not match with the number of supporting cells, which are present throughout entire ectoneural region of the radial nerve cords (Mashanov et al., 2016). It is possible that a subpopulation of the supporting cells express RGP, but it seems more likely that the cells expressing RGP in radial nerve cords are neurons. But as with supporting cells, the processes of neuronal cell bodies located in the ectoneural epithelium are confined to neuropile of the ectoneural region (Moore and Thorndyke, 1993; Mashanov et al., 2016), and therefore it is not clear how ectoneural neurons in the radial nerve cords expressing RGP could release RGP to act as a hormone. Furthermore, the overall pattern of RGP expression in the ambulacrum, with RGP‐expressing cells or groups of cells located along the length of the nerve cords and in the tube feet, is perhaps more consistent with a role in coordination of tube foot activity as opposed to being a source of RGP as a reproductive hormone. Thus, RGP‐expressing cells located in the radial nerve cords could be considered as functionally equivalent to neurons expressing relaxin‐3 that are located in the brainstem of mammals (Sherwood, 2004).

Further evidence that the radial nerve cords may not be the physiological source of GSS/RGP as a hormonal regulator of spawning is the observation that the concentration/expression of GSS/RGP in the radial nerve cords remains constant throughout the year (Chaet, 1966; Mita, 2013). This contrasts with the concentration of GSS/RGP in the coelomic fluid, which increases just prior to spawning (Kanatani and Ohguri, 1966; Kanatani and Shirai, 1969), providing the key evidence that GSS/RGP acts as a hormonal regulator of gamete maturation and release physiologically. If the radial nerve cords are not the physiological source of GSS/RGP as a gonadotropic hormone in starfish, then what is the source?

Here we report the novel discovery of RGP‐expressing cells located at the tips of each arm in *A. rubens*. A specialized anatomical feature of the arm tip is the terminal tentacle, which is similar in structure to the locomotory tube feet but has nonlocomotory sensory functions (Hennebert et al., 2013). At the base of the terminal tentacle is the pigmented optic cushion, which contains photoreceptive cells and functions as a simple eye enabling visual orientation (Penn and Alexander, 1980; Garm and Nilsson, 2014). AruRGP‐expressing cells were not observed in the optic cushion but, consistent with the expression of AruRGP in the podia of the locomotory tube feet, AruRGP‐expressing cells were evident in the terminal tentacle. Moreover, intense expression of AruRGP was observed in a band of cells located in the body wall epithelium that forms the “ceiling” and “walls” of a cavity surrounding the terminal tentacle and optic cushion. The discovery of these AruRGP‐expressing cells is fascinating because their location in the arm tips, in close association with sensory systems, make them good candidates as a physiological source of RGP for regulation of gamete release in response to environmental cues. Observation of these cells at high magnification reveals that they are neuron‐like, with stained processes emanating from stained cell bodies. Furthermore, consistent with a neuronal cell type, the AruRGP‐expressing cells in arm tip epithelium are underlain by a layer that is immunoreactive with antibodies to the neural‐specific protein synaptotagmin B. However, it remains to be determined where the processes of AruRGP‐expressing cells in the arm tip epithelium project to. To address this issue it may be necessary to generate antibodies to AruRGP so that the peptide itself, rather the just the mRNA encoding it, can visualized throughout the entirety of cells expressing AruRGP.

If the RGP‐expressing cells in the arm tips are the physiological source of RGP as a gonadotropic hormone, how in principle could the release of RGP from these cells into the coelomic fluid be triggered? A variety of environmental factors are thought to be important in triggering spawning in starfish (Mercier and Hamel, 2013), including increasing day length (Pearse et al., 1986; Byrne et al., 1997), changes in water temperature (Pearse and Walker, 1986), and the release of gametes by conspecifics (Hamel and Mercier, 1995). The RGP‐expressing cells in the arm tips are ideally positioned to detect and integrate such environmental cues, located as they are in the arm tip body wall epithelium and in close proximity to the sensory terminal tentacle and optic cushion. Identification of these cells provides a basis for experimental studies in which their role as putative sources of RGP as a gonadotropic hormone in starfish could be investigated.

## CONFLICT OF INTEREST

The authors declare that they have no conflicts of interest.

## ROLES OF AUTHORS

All authors had full access to all the data in the study and take responsibility for the integrity of the data and the accuracy of the data analysis. Study concept and design: ML, MM, and MRE. Acquisition of data: ML (cDNA cloning and generation of probes for AruRGP precursor; preparation of sections of starfish; mRNA in situ hybridization, MM (testing bioactivity of AruRGP and PpeRGP), ME (development of methodology for mRNA in situ hybridisation in starfish), CGZ and AMJ (mass spectrometry). Analysis and interpretation of data: ML, MM, CGZ, AMJ, and MRE. Drafting of the article: ML, MM, CGZ, AMJ, and MRE. Obtained funding: MRE, MM, and AMJ. Technical and material support: ME. Study supervision: MRE.

## References

[cne24141-bib-0001] Bathgate RA , Samuel CS , Burazin TC , Layfield S , Claasz AA , Reytomas IG , Dawson NF , Zhao C , Bond C , Summers RJ , Parry LJ , Wade JD , Tregear GW . 2002 Human relaxin gene 3 (H3) and the equivalent mouse relaxin (M3) gene. Novel members of the relaxin peptide family. J Biol Chem 277:1148–1157. 11689565 10.1074/jbc.M107882200

[cne24141-bib-0002] Brown MR , Clark KD , Gulia M , Zhao Z , Garczynski SF , Crim JW , Suderman RJ , Strand MR . 2008 An insulin‐like peptide regulates egg maturation and metabolism in the mosquito *Aedes aegypti* . Proc Natl Acad Sci U S A 105:5716–5721. 18391205 10.1073/pnas.0800478105 PMC2311378

[cne24141-bib-0003] Burke RD , Osborne L , Wang D , Murabe N , Yaguchi S , Nakajima Y . 2006 Neuron‐specific expression of a synaptotagmin gene in the sea urchin *Strongylocentrotus purpuratus* . J Comp Neurol 496:244–251. 16538680 10.1002/cne.20939

[cne24141-bib-0004] Byrne M , Morrice M , Wolf B . 1997 Introduction of the northern Pacific asteroid *Asterias amurensis* to Tasmania: reproduction and current distribution. Mar Biol 127:673–685.

[cne24141-bib-0005] Caine GD , Burke RD . 1985 Immunohistochemical localisation of gonad stimulating substance in the seastar *Pycnopodia helianthoides* In: Keegan BF, O'Connor BDS, eds. Proceedings of the Fifth international Echinoderm Conference. Galway, Ireland: Balkema, Rotterdam p 495–498.

[cne24141-bib-0006] Chaet AB . 1964 A mechanism for obtaining mature gametes from starfish. Biol Bull 126:8–13.

[cne24141-bib-0007] Chaet AB 1966 The gamete‐shedding substances of starfishes: a physiological‐biochemical study. Am Zool 6:263–271. 5933237 10.1093/icb/6.2.263

[cne24141-bib-0008] Chaet A , McConnaughy R . 1959 Physiologic activity of nerve extracts. Biol Bull 117:407–408.

[cne24141-bib-0009] Chiu AY , Hunkapiller MW , Heller E , Stuart DK , Hood LE , Strumwasser F . 1979 Purification and primary structure of the neuropeptide egg‐laying hormone of *Aplysia californica* . Proc Natl Acad Sci U S A 76:6656–6660. 293751 10.1073/pnas.76.12.6656 PMC411927

[cne24141-bib-0010] Conn PJ , Kaczmarek LK . 1989 The bag cell neurons of *Aplysia* . Mol Neurobiol 3:237–273. 2698177 10.1007/BF02740607

[cne24141-bib-0011] Felsenstein J . 1985 Confidence limits on phylogenies: an approach using the bootstrap. Evolution 783–791. 10.1111/j.1558-5646.1985.tb00420.x 28561359

[cne24141-bib-0012] Garm A , Nilsson DE . 2014 Visual navigation in starfish: first evidence for the use of vision and eyes in starfish. Proc Biol Sci 281:20133011. 24403344 10.1098/rspb.2013.3011 PMC3896028

[cne24141-bib-0013] Gotze M , Pettelkau J , Schaks S , Bosse K , Ihling CH , Krauth F , Fritzsche R , Kuhn U , Sinz A . 2012 StavroX—a software for analyzing crosslinked products in protein interaction studies. J Am Soc Mass Spectrom 23:76–87. 22038510 10.1007/s13361-011-0261-2

[cne24141-bib-0014] Hamel J‐F , Mercier A . 1995 Prespawning behavior, spawning, and development of the brooding starfish *Leptasterias polaris* . Biol Bull 188:32–45. 10.2307/1542065 29281304

[cne24141-bib-0015] Haraguchi S , Ikeda N , Abe M , Tsutsui K , Mita M . 2016 Nucleotide sequence and expression of relaxin‐like gonad‐stimulating peptide gene in starfish *Asterina pectinifera* . Gen Comp Endocrinol 227:115–119. 26166482 10.1016/j.ygcen.2015.06.017

[cne24141-bib-0016] Hennebert E , Jangoux M , Flammang P . 2013 Functional biology of asteroid tube feet In: Lawrence J, ed. Starfish: Biology and ecology of the Asteroidea. Baltimore, MD: Johns Hopkins University Press p 24–36.

[cne24141-bib-0017] Hisaw FL . 1926 Experimental relaxation of the pubic ligament of the guinea pig. Exp Biol Med 23:661–663.

[cne24141-bib-0018] Hsu SY , Semyonov J , Park JI , Chang CL . 2005 Evolution of the signaling system in relaxin‐family peptides. Ann N Y Acad Sci 1041:520–529. 15956755 10.1196/annals.1282.078

[cne24141-bib-0019] Kanatani H . 1979 Hormones in echinoderms In: Barrington E, ed. Hormones and evolution. Vol 1 London: Academic Press p 273–307.

[cne24141-bib-0020] Kanatani H , Ohguri M . 1966 Mechanism of starfish spawning. I. Distribution of active substance responsible for maturation of oocytes and shedding of gametes. Biol Bull 131:104–114.

[cne24141-bib-0021] Kanatani H , Shirai H . 1969 Mechanism of starfish spawning. II. Some aspects of action of a neural substance obtained from radial nerve. Biol Bull 137:297–311.

[cne24141-bib-0022] Kanatani H , Shirai H . 1970 Mechanism of starfish spawning. 3. Properties and action of meiosis‐inducing substance produced in gonad under influence of gonad‐stimulating substance. Dev Growth Differ 12:119–140. 5530286 10.1111/j.1440-169x.1970.00119.x

[cne24141-bib-0023] Kessner D , Chambers M , Burke R , Agus D , Mallick P . 2008 ProteoWizard: open source software for rapid proteomics tools development. Bioinformatics 24:2534–2536. 18606607 10.1093/bioinformatics/btn323 PMC2732273

[cne24141-bib-0024] Kumar S , Stecher G , Tamura K . 2016 MEGA7: Molecular evolutionary genetics analysis version 7.0 for Bigger datasets. Mol Biol Evol 33:1870–1874. 27004904 10.1093/molbev/msw054 PMC8210823

[cne24141-bib-0025] Mashanov V , Zueva O , Rubilar T , Epherra L , García‐Arrarás J . 2016 Echinodermata In: Schmidt‐Rhaesa A, Harzsch S, Purschke G, eds. Structure and evolution of invertebrate nervous systems. Oxford, UK: Oxford University Press p 665–688.

[cne24141-bib-0026] Mercier A , Hamel J‐F . 2013 Reproduction in Asteroidea In: Lawrence J, ed. Starfish: Biology and ecology of the Asteroidea. Baltimore, MD: Johns Hopkins University Press p 37–50.

[cne24141-bib-0027] Mita M . 2013 Relaxin‐like gonad‐stimulating substance in an echinoderm, the starfish: a novel relaxin system in reproduction of invertebrates. Gen Comp Endocrinol 181:241–245. 22841765 10.1016/j.ygcen.2012.07.015

[cne24141-bib-0028] Mita M , Katayama H . 2016 A relaxin‐like gonad‐stimulating peptide from the starfish *Aphelasterias japonica* . Gen Comp Endocrinol 229:56–61. 26944483 10.1016/j.ygcen.2016.02.025

[cne24141-bib-0029] Mita M , Ito C , Kubota E , Nagahama Y , Shibata Y . 2009a Expression and distribution of gonad‐stimulating substance in various organs of the starfish *Asterina pectinifera* . Ann N Y Acad Sci 1163:472–474. 19456390 10.1111/j.1749-6632.2008.03626.x

[cne24141-bib-0030] Mita M , Yoshikuni M , Ohno K , Shibata Y , Paul‐Prasanth B , Pitchayawasin S , Isobe M , Nagahama Y . 2009b A relaxin‐like peptide purified from radial nerves induces oocyte maturation and ovulation in the starfish, *Asterina pectinifera* . Proc Natl Acad Sci U S A 106:9507–9512. 19470645 10.1073/pnas.0900243106 PMC2685251

[cne24141-bib-0031] Mita M , Daiya M , Haraguchi S , Tsutsui K , Nagahama Y . 2015 A new relaxin‐like gonad‐stimulating peptide identified in the starfish *Asterias amurensis* . Gen Comp Endocrinol 222:144–149. 26163025 10.1016/j.ygcen.2015.07.002

[cne24141-bib-0032] Moore SJ , Thorndyke MC . 1993 Immunocytochemical mapping of the novel echinoderm neuropeptide SALMFamide 1 (S1) in the starfish *Asterias rubens* . Cell Tissue Res 274:605–618. 8293452 10.1007/BF00314559

[cne24141-bib-0033] Nesvizhskii AI , Keller A , Kolker E , Aebersold R . 2003 A statistical model for identifying proteins by tandem mass spectrometry. Anal Chem 75:4646–4658. 14632076 10.1021/ac0341261

[cne24141-bib-0034] Pearse JS , Walker CW . 1986 Photoperiodic regulation of gametogenesis in a North Atlantic sea star, *Asterias vulgaris* . Int J Invert Reprod Dev 9:71–77.

[cne24141-bib-0035] Pearse JS , Eernisse DJ , Pearse VB , Beauchamp KA . 1986 Photoperiodic regulation of gametogenesis in sea stars, with evidence for an annual calendar independent of fixed daylength. Am Zool 26:417–431.

[cne24141-bib-0036] Penn P , Alexander C . 1980 Fine structure of the optic cusion in the asteroid *Nepanthia belcheri* . Mar Biol 58:251–256.

[cne24141-bib-0037] Pentreath VW , Cobb JL . 1972 Neurobiology of echinodermata. Biol Rev Camb Philos Soc 47:363–392. 4563848 10.1111/j.1469-185x.1972.tb00977.x

[cne24141-bib-0038] Pierce JG , Parsons TF . 1981 Glycoprotein hormones: structure and function. Annu Rev Biochem 50:465–495. 6267989 10.1146/annurev.bi.50.070181.002341

[cne24141-bib-0039] Saha AK , Tamori M , Inoue M , Nakajima Y , Motokawa T . 2006 NGIWYamide‐induced contraction of tube feet and distribution of NGIWYamide‐like immunoreactivity in nerves of the starfish *Asterina pectinifera* . Zoolog Sci 23:627–632. 16908962 10.2108/zsj.23.627

[cne24141-bib-0040] Saitou N , Nei M . 1987 The neighbor‐joining method: a new method for reconstructing phylogenetic trees. Mol Biol Evol 4:406–425. 3447015 10.1093/oxfordjournals.molbev.a040454

[cne24141-bib-0041] Schwabe C , McDonald JK . 1977 Relaxin: a disulfide homolog of insulin. Science 197:914–915. 887933 10.1126/science.887933

[cne24141-bib-0042] Semmens DC , Mirabeau O , Moghul I , Pancholi MR , Wurm Y , Elphick MR . 2016 Transcriptomic identification of starfish neuropeptide precursors yields new insights into neuropeptide evolution. Open Biol 6:150224. 26865025 10.1098/rsob.150224 PMC4772807

[cne24141-bib-0043] Sherwood OD . 2004 Relaxin's physiological roles and other diverse actions. Endocr Rev 25:205–234. 15082520 10.1210/er.2003-0013

[cne24141-bib-0044] Shirai H . 1986 Gonad‐stimulating and maturation‐inducing substance. Methods Cell Biol 27:73–88. 3517589 10.1016/s0091-679x(08)60343-x

[cne24141-bib-0045] Smith CM , Ryan PJ , Hosken IT , Ma S , Gundlach AL . 2011 Relaxin‐3 systems in the brain—the first 10 years. J Chem Neuroanat 42:262–275. 21693186 10.1016/j.jchemneu.2011.05.013

[cne24141-bib-0046] Thompson JD , Higgins DG , Gibson TJ . 1994 CLUSTAL W: improving the sensitivity of progressive multiple sequence alignment through sequence weighting, position‐specific gap penalties and weight matrix choice. Nucl Acids Res 22:4673–4680. 7984417 10.1093/nar/22.22.4673 PMC308517

[cne24141-bib-0047] Unger H . 1962 Experimentelle und histologische Untersuchungen über Wirkfaktoren aus dem Nervensystem von *Asterias (Marthasterias) glacialis* (Asteroidea; Echinodermata). Zool Jb Abt Allg Zool Physiol Tiere 69:481–536.

[cne24141-bib-0048] Zuckerkandl E , Pauling L . 1965 Evolutionary divergence and convergence in proteins. Evolv Genes Prot 97:97–166.

